# The ubiquitous catechol moiety elicits siderophore and angucycline production in *Streptomyces*

**DOI:** 10.1038/s42004-022-00632-4

**Published:** 2022-02-03

**Authors:** Doris A. van Bergeijk, Somayah S. Elsayed, Chao Du, Isabel Nuñez Santiago, Anna M. Roseboom, Le Zhang, Victor J. Carrión, Herman P. Spaink, Gilles P. van Wezel

**Affiliations:** 1 Institute of Biology, Leiden University, Sylviusweg 72, 2333 BE Leiden, The Netherlands; 2 Netherlands Institute of Ecology, NIOO-KNAW, Droevendaalsesteeg 10, 6708 PB Wageningen, The Netherlands

**Keywords:** Microbiology, Chemical ecology, Biosynthesis, Metabolomics

## Abstract

Actinobacteria are a rich source of bioactive molecules, and genome sequencing has shown that the vast majority of their biosynthetic potential has yet to be explored. However, many of their biosynthetic gene clusters (BGCs) are poorly expressed in the laboratory, which prevents discovery of their cognate natural products. To exploit their full biosynthetic potential, better understanding of the signals that promote the expression of BGCs is needed. Here, we show that the human stress hormone epinephrine (adrenaline) elicits siderophore production by Actinobacteria. Catechol was established as the likely eliciting moiety, since similar responses were seen for catechol and for the catechol-containing molecules dopamine and catechin but not for related molecules. Exploration of the catechol-responsive strain *Streptomyces* sp. MBT84 using mass spectral networking revealed elicitation of a BGC that produces the angucycline glycosides aquayamycin, urdamycinone B and galtamycin C. Heterologous expression of the catechol-cleaving enzymes catechol 1,2-dioxygenase or catechol 2,3-dioxygenase counteracted the eliciting effect of catechol. Thus, our work identifies the ubiquitous catechol moiety as a novel elicitor of the expression of BGCs for specialized metabolites.

## Abstract (web summary)

The biosynthetic gene clusters of Actinobacteria are poorly expressed in the laboratory, necessitating a better understanding of the signals that promote their expression in order to exploit their full biosynthetic potential. Here, the authors show that the stress hormone epinephrine elicits the expression of biosynthetic gene clusters and influences the metabolism of *Streptomyces*, with the catechol moiety proving key to this response.

## Introduction

The phylum Actinobacteria represents a highly diverse group of bacteria with extraordinary metabolic potential^1^. Their specialized metabolites include most of the clinically used antibiotics along with numerous cancer chemotherapeutics, immunosuppressants, and pesticides, and are therefore of great importance for application as clinical drugs or in agriculture^2^. This metabolic versatility makes Actinobacteria attractive sources for drug discovery, for which there is an urgent need due to the global rise of drug resistance^3,4^. Traditional high-throughput screening suffers from low return on investments due to dereplication, in other words, the rediscovery of bioactive compounds that have been identified before^5,6^. Next-generation sequencing technologies revealed a huge repository of previously unseen biosynthetic gene clusters (BGCs) in Actinobacteria, which showed that their potential as producers of bioactive molecules had been grossly underestimated^1,7,8^. However, these BGCs are often not expressed under laboratory conditions, most likely because the environmental cues that activate their expression in their original habitat are missing^9,10^. Indeed, molecules of actinobacterial origin continue to be discovered that have important new structural and/or functional features, for example the antifungal cyphomycin^11^_,_ the glycopeptide antibiotic corbomycin^12^, and the angucycline-derived polyketide lugdunomycin^13^. To more efficiently exploit Actinobacteria as a resource of chemical diversity, we need to first understand the triggers and cues that promote their expression. This knowledge can then be translated to eliciting approaches to activate the expression of BGCs and produce their cognate bioactive compounds at high throughput^14^.

One approach to identify the cues that activate BGC expression lies in understanding the ecological context of specialized metabolite production^14^. Actinobacteria inhabit a wide range of terrestrial and aquatic ecosystems^9^. Within these environments, specialized metabolites play an important role in survival through the mediation of resource competition^15,16^, protection against oxidative stresses^17^ and uptake of essential nutrients^18^. This requires careful timing of production and it is therefore likely that environmental signals indicative of specific stresses (such as nutrient levels and the presence of competitors) have been incorporated in the regulation of BGC expression^19–21^. Indeed, nutrient availability and co-culture of Actinobacteria with other microorganisms significantly influence their specialized metabolite production^22,23^.

Although often described as free-living organisms, Actinobacteria live in and around a wide variety of other organisms, including higher eukaryotes such as plants, insects, marine organisms, and mammals^11,14^. As part of the microbiomes of these hosts, Actinobacteria are exposed to host-associated signaling molecules, many of which will likely influence their specialized metabolism^14^. Indeed, plant stress hormones, such as salicylic acid and jasmonic acid, have been shown to increase the antibiotic activity of endophytic streptomycetes^24^. These hormones are excreted by plants under pathogenic stress, and their release might represent a “cry for help” through which the plant may activate the production of bioactive substances by members of their microbiome, in order to counteract a pathogenic attack^24^. Besides plant hormones, also animal stress hormones have been shown to influence bacteria. For example, the human opioid dynorphin stimulates the production of pyocyanin in *Pseudomonas aeruginosa*^25^. Specifically, catecholamines, which include the well-known ‘fight or flight’ hormone adrenaline (also known as epinephrine), influence bacterial growth^26,27^, biofilm formation^28^, and horizontal gene transfer^29^. We hypothesize that animal stress hormones may play a role in the control of antibiotic production of Actinobacteria.

In this work, we demonstrate that the animal stress hormone epinephrine can influence the specialized metabolism of streptomycetes, specifically the siderophore production. The catechol moiety showed to be key to this response and this finding could be translated to other host-specific compounds (human and plant) containing a catechol moiety. Catechol by itself enhanced the production of angucyclines. Taken together, these results illustrate that catechol, by itself and as part of plant- and animal-associated molecules, can serve as elicitor of specialized metabolism.

## Results

### Epinephrine alters antibiotic production of Actinobacteria

Actinobacteria live in close association with higher eukaryotes, such as plants and animals. Host stress molecules might play a role in the regulation of actinobacterial specialized metabolism, perhaps reflecting a “cry for help” from host to bacterium^24^. We, therefore, investigated if animal stress hormones can affect the growth and metabolism of Actinobacteria. For this, we analyzed the effect of epinephrine, also known as adrenaline, on a selection of our in-house actinobacterial strain collection that was previously shown to require particular growth conditions for the production of antibiotics^30^. We used the Gram-positive *Bacillus subtilis* 168 and the Gram-negative *Escherichia coli* ASD19 as indicator strains. Minimal Medium agar (MM) and Nutrient Agar (NA) plates with or without 100 µM epinephrine (bitartrate salt) or tartaric acid (as control for the added bitartrate) were inoculated with spots from spore stocks of different actinobacterial strains. The chosen concentration of 100 µM was based on literature^27^. To see if the addition of epinephrine to the growth media affected the susceptibility of the indicator strains against antibiotics, we added a diffusion disc with ampicillin (6 µg) to each plate and tested whether the presence of epinephrine in the growth medium affected the size of the inhibition zone of ampicillin against *E. coli* and *B. subtilis*. No differences were observed, confirming that the addition of epinephrine to the growth medium did not affect the susceptibility of the indicator strains.

On MM, only a small number of strains showed a change in bioactivity in response to epinephrine (Fig. S1A). Both promotion and inhibition of bioactivity were observed, of which a decrease in bioactivity was observed most frequently (often against both *E. coli* and *B. subtilis*). For some strains, this decrease in bioactivity could be linked to inhibition of growth in presence of epinephrine. On NA, no significant changes in bioactivity were observed except for the elicitation of a large semi-transparent halo surrounding *Streptomyces* sp. MBT42 by epinephrine (Fig. S1B).

### The catechol moiety is key to the eliciting effect of epinephrine on *Streptomyces* sp. MBT42

**Fig. 1:**
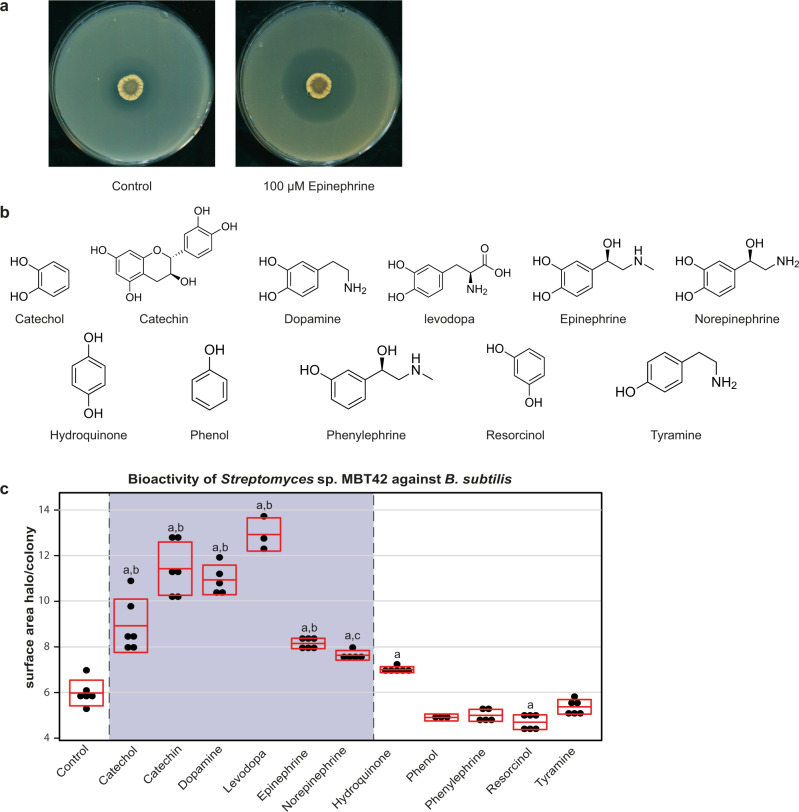
The catechol-moiety is key to the eliciting effect of epinephrine on *Streptomyces* sp. MBT42. **a** *Streptomyces* sp. MBT42 was grown on NA supplemented with and without 100 µM epinephrine (*n* = 3). After 4 days of growth, plates were overlaid with *B. subtilis* to test for antimicrobial activity. Note the increased semi-transparent halo surrounding *Streptomyces* sp. MBT42 grown in presence of epinephrine. **b** An overview of the structurally related compounds tested. **c** Effect of different compounds on the bioactivity of *Streptomyces* sp. MBT42 against *B. subtilis*. Bioactivity was quantified by measuring the ratio between the surface area of the inhibition zone and the colony (data represent two independent experiments, the number of biologically independent replicates (=*n*) for each group is indicated in the figure, mean and standard deviation are indicated in red, the grey box highlights the response to compounds with a catechol moiety). One-way ANOVA, followed by a *post hoc* Tukey’s honest significant difference (HSD) test, was performed to compare the difference in bioactivity between the growth conditions. The symbols indicate a significant increase in bioactivity compared to (**a**) the control, (**b**) to compounds lacking a catechol moiety, and (**c**) to compounds lacking a catechol moiety except hydroquinone.

To analyze the eliciting effect of epinephrine in more detail, we selected *Streptomyces* sp. MBT42, as this strain reproducibly showed a strong response to the hormone when the strain was grown on NA agar plates (Fig. 1a). In particular, we were interested to see whether epinephrine itself or any of its chemical constituents would be responsible for the eliciting effect. To do so, the effects of different catecholamines and structurally related compounds were assessed (Fig. 1b). Besides epinephrine, we also included catechin, dopamine, levodopa, and norepinephrine. Interestingly, all of these compounds had an effect similar to or stronger than epinephrine and significantly elicited *Streptomyces* sp. MBT42 compared to control. Since all of the tested compounds contain a catechol moiety, we wondered if catechol (1,2-dihydroxybenzene) may be the moiety primarily responsible for the observed eliciting effect. We, therefore, tested the effect of catechol itself, and as controls we used several structural analogues, namely the *meta* isomer resorcinol (1,3-dihydroxybenzene) and the *para* isomer hydroquinone (1,4-dihydroxybenzene), as well as molecules containing a monohydroxy-substituted benzene ring, such as phenol, phenylephrine, and tyramine. Importantly, of these compounds, catechol itself significantly enhanced antibiotic production by *Streptomyces* sp. MBT42 compared to control, while most other compounds failed to elicit antibiotic production. Hydroquinone had a mild eliciting effect, similar to that of norepinephrine (Fig. 1c), However, this effect was significantly lower compared to the effect of the compounds with a catechol-moiety such as epinephrine and catechol. These data strongly suggest that the catechol moiety is key to the response of *Streptomyces* sp. MBT42 to the stress hormone epinephrine.

### Catechol compounds enhance siderophore production in *Streptomyces* sp. MBT42

**Fig. 2:**
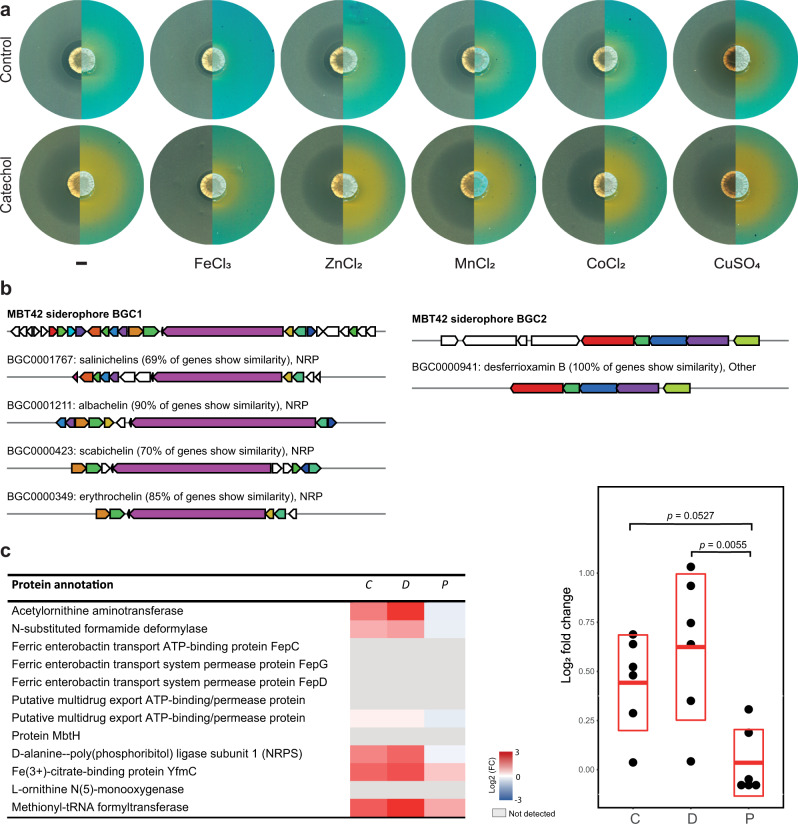
Catechols elicit siderophore production by *Streptomyces* sp. MBT42. **a** *Streptomyces* sp. MBT42 was grown on NA with and without 100 µM catechol, supplemented with various metal salts (5 µM) (*n* = 3). After 4 days of growth, plates were overlaid with *B. subtilis* (left) to test for antimicrobial activity or with CAS agar to detect the extracellular production of iron-chelating molecules (orange halos; right). Note that catechol inhibits the growth of *B. subtilis* and induces siderophore production, and that these zones are highly comparable. When iron was added to the medium, siderophore production was almost completely inhibited and the semi-transparent halo was no longer visible. **b** Analysis using antiSMASH revealed two siderophore BGCs in the genome of MBT42. KnownClusterBlast output from antiSMASH which shows similar clusters from the MIBiG database. Genes marked with the same color are interrelated; white genes have no relationship. **c** Change in protein expression of the salinichelin-like BGC of *Streptomyces* sp. MBT42 in response to catechin (C), dopamine (D), and phenylephrine (P). The heatmap shows the log_2_ fold change of protein level compared to control (*n* = 4). Note the similarity between catechin and dopamine. The scatter plot shows the average log_2_ fold change of each protein. The mean and standard deviation are indicated in red The average log_2_ fold changes in protein level were compared between the different groups by one-way ANOVA, followed by a post hoc Tukey’s HSD test.

The catechol moiety is well known for its iron-chelating properties. For example, catechin is a strong siderophore^31^. We therefore hypothesized that the addition of catechol-containing molecules might result in lower iron availability, which may trigger siderophore production. To test this hypothesis, we spotted MBT42 spores onto NA with or without 100 µM catechol, and overlaid the plates with CAS agar solution after 4 days of growth. An orange halo was formed that is indicative of siderophore production, which was strongly increased in the presence of catechol (Fig. 2a). These siderophore-related halos matched the semi-transparent halos that were observed when *Streptomyces* sp. MBT42 was overlaid with *B. subtilis*.

Siderophore production is suppressed by iron availability. We, therefore, tested whether the addition of iron could compensate for the effect of catechol on *Streptomyces* sp. MBT42 Indeed, when 5 µM FeCl_3_ was added to the medium, siderophore production was strongly reduced, and this coincided with the disappearance of the semi-transparent halo (Fig. 2a). The same result was obtained when FeSO_4_ was added. Other metal ions did not have this effect (Fig. 2a).

We then wondered which siderophore may be produced by *Streptomyces* sp. MBT42. The full genome of *Streptomyces* sp. MBT42 was sequenced (GenBank accession number: JAJNOJ000000000) (Table S3) and the natural product BGCs predicted using antiSMASH 6.0^32^. This revealed two candidate BGCs for siderophores; one BGC was identical to the desferrioxamine BGC and one BGC showed high similarity to that for salinichelin and albachelin (Fig. 2b). To find out which siderophore BGC was induced by catechol, we used MS-based quantitative proteomic analysis, which is an efficient way to establish changes in expression of BGCs in response to eliciting signals^33,34^. The proteome samples of *Streptomyces* sp. MBT42 grown on NA agar plates with and without 100 µM dopamine, catechin, or phenylephrine were compared. Dopamine and catechin were chosen, because these compounds induced a similarly strong response despite their structural differences, while phenylephrine was added as a control, since this compound lacks a catechol moiety. After five days of growth, the biomass was harvested and snap-frozen in liquid nitrogen. Subsequent quantitative proteomic analysis was performed on four replicate samples per growth condition. None of the proteins of the desferrioxamine BGC were detected under the chosen conditions, nor desferrioxamine itself, indicating that this BGC is not expressed. Importantly, biosynthetic proteins of the salinichelin-like BGC showed significantly increased expression in dopamine-grown cultures as compared to phenylephrine-grown cultures (*p*-value < 0.05) (Fig. 2c). The increase in protein levels in response to catechin followed a trend similar to dopamine, and was not observed in phenylephrine-grown cultures (Fig. 2c). Taken together, this shows that catechol compounds act as an elicitor of siderophore biosynthesis, which is most likely explained by the sequestering of iron.

### Catechol elicits the production of bioactive specialized metabolites

**Fig. 3:**
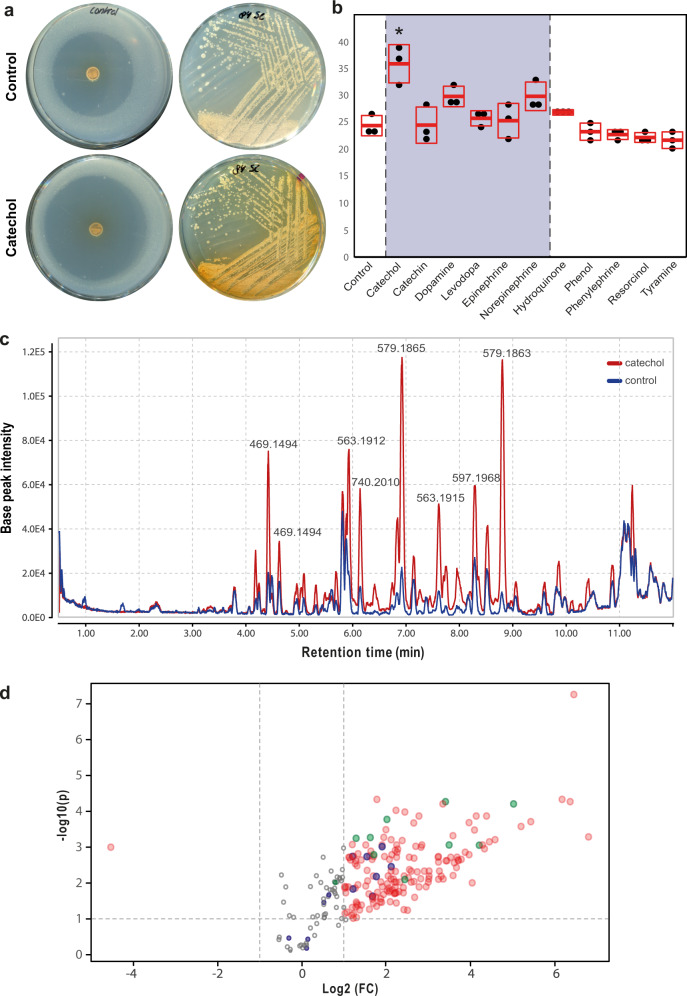
Catechol elicits the bioactivity and metabolite production of *Streptomyces* sp. MBT84. **a** The antibacterial activity against *B. subtilis* 168 and yellow pigment production of *Streptomyces* sp. MBT84 are increased in the presence of catechol. **b** None of the structurally related compounds, including epinephrine, increased the antibiotic production by *Streptomyces* sp. MBT84. One-way ANOVA, followed by a post hoc Tukey’s HSD test, was performed to compare the difference in bioactivity between the growth conditions (*p < 0.001, *n* = 3, mean and standard deviation are indicated in red, the grey box highlights the response to compounds with a catechol moiety). **c** LC-MS chromatogram overlay of the crude extract of *Streptomyces* sp. MBT84 grown with and without catechol. Multiple peaks were increased in intensity in the presence of catechol. **d** Volcano plot highlighting the increased metabolite production by *Streptomyces* sp. MBT84 in response to catechol. The *x* and *y* axes represent the log_2_ fold changes and the corresponding −log_10_ FDR-adjusted *p*-value of all the mass features, respectively. Red circles represent the mass features in catechol-grown cultures with a significant intensity difference of more than twofold compared to control cultures (FDR-adjusted *p* ≤ 0.1). Circles situated in the top left and top right quadrants represent the mass features that are most induced or repressed, respectively, by catechol with high statistical significance. The *m/z* values 469.149 and 579.186 are shown in purple and green, respectively (*n* = 6).

The specificity of the above-described response to the catechol moiety suggested that catechol may be applied as a general elicitor of natural product biosynthesis in Actinobacteria. We, therefore, tested the effect of catechol on some of the well-characterized Actinobacteria in our collection. Again, we observed both promotion and inhibition of bioactivity in the presence of catechol (Fig. S2). *Streptomyces* sp. MBT84 responded particularly well to catechol, reproducibly producing strong antibacterial activity against *B. subtilis* in response to catechol (Fig. 3a). The bioactivity coincided with enhanced production of a yellow/brown pigment (Fig. 3a). However, in this case none of the structurally related compounds, including epinephrine, increased antibiotic production (Fig. 3b). Also iron did not affect the changes in bioactivity and pigment production and no siderophore production was observed in the CAS agar overlay. Thus we ruled out that siderophore production was the cause of the observed growth inhibition.

To identify the nature of the metabolites produced by *Streptomyces* sp. MBT84 in response to catechol, the strain was streaked on MM agar plates and the metabolites were then extracted from the spent agar after five days of growth using EtOAc. The crude extracts of the catechol-grown cultures showed increased bioactivity as compared to those grown under control conditions, while media blanks showed no activity. To rule out that catechol potentiates the bioactivity, we tested whether the presence of catechol in the growth medium affected the size of the inhibition zone of different antibiotics and the crude extracts of MBT84 against *B. subtilis*. No differences were observed (Fig. S3), which shows that the addition of catechol to the growth medium does not affect the susceptibility of the indicator strains against antibiotics, nor does it potentiate the activity of the crude extracts.

Liquid chromatography-mass spectrometry (LC-MS) analysis revealed multiple peaks in the chromatogram that were substantially overrepresented in the samples obtained from the catechol-grown cultures (Fig. 3c). The metabolomic profile of *Streptomyces* sp. MBT84 grown with and without catechol was compared through a volcano plot generated using MetaboAnalyst^35^ (Fig. 3d). In the volcano plot, the majority of the mass features were significantly upregulated in catechol-grown cultures (FC > 2, FDR-adjusted *p*-value < 0.1). The most notable ones were those with *m/z* values 469.1494 and 579.1864, which represented several highly intense mass features sharing the same exact masses but with different retention times. In addition, multiple in-source fragments were among the mass features which were significantly upregulated with catechol. The mass features related to *m/z* 469.1494 and 579.1864 are likely due to yellowish-colored metabolites, since they showed in the ultraviolet (UV) chromatogram corresponding peaks having UV absorption maxima of 420–440 nm. This corresponds well to the observed increase in yellow pigmentation when *Streptomyces* sp. MBT84 was grown in the presence of catechol.

**Fig. 4:**
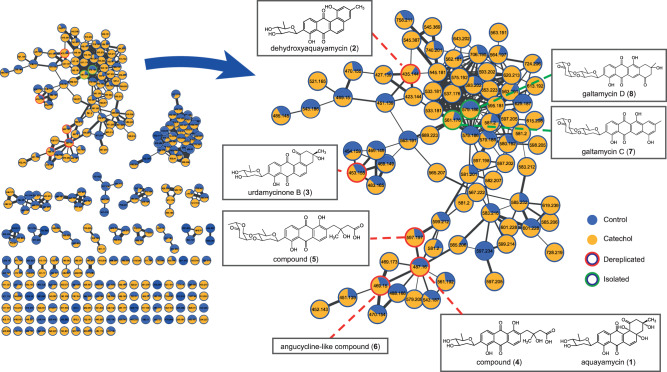
Molecular network of the ions detected in the crude extracts of *Streptomyces* sp. MBT84 revealing a large spectral family of angucyline compounds elicited by catechol. A pie chart was mapped to the nodes which represents the abundance of each m/z value in the control (blue) and catechol-grown crude extracts (yellow). Nodes highlighted in red represent dereplicated metabolites, while those highlighted in green represent the compounds isolated and identified in this study. *Streptomyces* sp. MBT84 was grown for five days on MM agar plates with or without 100 µM catechol (*n* = 6).

To gain more insight into which metabolites were induced by catechol and whether these were structurally related, molecular networking was employed using the Global Natural Products Social molecular networking (GNPS) web tool. This generates a network wherein molecules with related scaffolds cluster together^36^. Due to the presence of multiple isomeric metabolites, we used the classical molecular networking workflow while enabling the MSCluster algorithm^36,37^. This allows to merge all mass features having similar MS and MS/MS spectra into one node, regardless of their different retention times. A network representing the ions detected in the crude extract of *Streptomyces* sp. MBT84 grown with and without catechol was constructed, revealing 258 nodes clustered in 21 spectral families (Fig. 4). Within the biggest spectral family, dereplication based on matching MS/MS spectra against the GNPS spectral library annotated several *m/z* values as being known metabolites belonging to the class of angucyclines, namely aquayamycin (**1**), dehydroxyaquayamycin (**2**), and urdamycinone B (**3**). In addition, two structurally related compounds (**4,5**) as well as an angucycline-like compound (**6**), were annotated. This angucyclines spectral family was more abundant in the presence of catechol (Fig. 4).

To ascertain that this network indeed consisted of angucycline glycoside-like compounds, we isolated the molecules with *m/z* 561.1760 (**7**) and 579.1866 (**8**). For this, *Streptomyces* sp. MBT84 was grown confluently on 12 × 12 mm MM agar plates supplemented with catechol and the metabolites were extracted with EtOAc. Following chromatographic isolation, **7** (red amorphous powder) and **8** (yellow amorphous powder) were obtained. The final structures of **7** and **8** were elucidated using nuclear magnetic resonance spectroscopy (NMR) (Supplemental data). Based on that, **7** was identified as galtamycin C, which has previously been isolated from an intertidal sediments-derived *Streptomyces* sp.^38^. As for **8**, it was found to be 1-oxo-3-hydroxy-3,4-dihydro-2*H*-galtamycin C based on the different 2D NMR correlations observed in comparison with **7**, together with the molecular formula and degrees of unsaturation obtained from its accurate mass (Supplementary Figs. S7–S23). To our knowledge **8** has not been previously described, and was thus designated as galtamycin D. Taken together, our data show that catechol enhances the biosynthesis of angucycline glycosides in *Streptomyces* sp. MBT84.

### Identification of the angucycline glycoside BGC in *Streptomyces* sp. MBT84

**Fig. 5:**
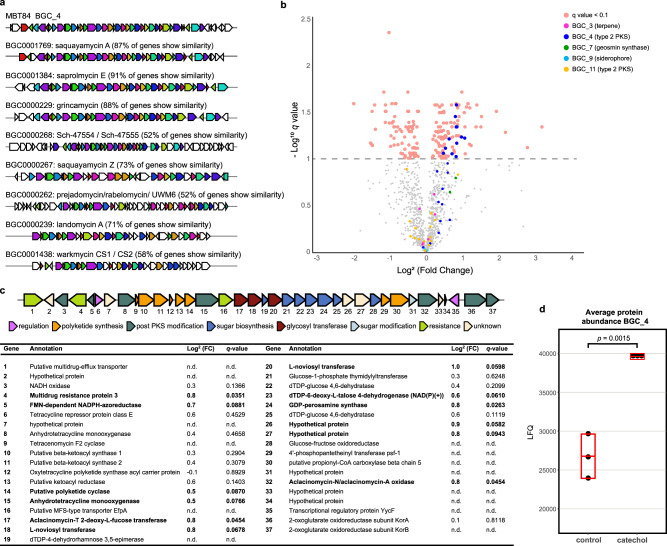
Identification of the BGC responsible for the production of angucycline glycosides in *Streptomyces* sp. MBT84 using antiSMASH and MS-based quantitative proteomics. **a** KnownClusterBlast output from antiSMASH which shows similar clusters from the MIBiG database. Genes marked with the same color are interrelated; white genes have no relationship. **b** Volcano plot of MS-based quantitative proteomics for cultures grown with and without catechol (*n* = 3). Proteins with an FDR-adjusted *p*-value ≥ 0.1 are grayed out. Proteins with a positive log_2_ fold change are higher expressed in catechol-grown cultures. **c** BGC4 coding for the biosynthesis of angucycline glycosides. Annotations are based on BLAST homology searches and genes are color-coded based on putative function. Significantly differentially expressed proteins are depicted in bold (FDR-adjusted *p* < 0.1, two-sample *t*-test, *n* = 3). **d** Average protein level of BGC4 of cultures grown with and without catechol (*n* = 3). Mean and standard deviation are indicated in red. Two sample *t*-test was done showing BGC4 is significantly differentially expressed in presence of catechol.

To identify the gene cluster responsible for the biosynthesis of the angucycline glycosides in *Streptomyces* sp. MBT84, we obtained its full genome sequence using PacBio sequencing (GenBank accession number: JAHTGP000000000). A draft genome was assembled resulting in 3 contigs (Table S3). Analysis using antiSMASH 6.0 revealed 21 putative BGCs (Table S4) of which BGC4 shows high similarity to the saquayamycin A BGC, and was therefore most likely responsible for the production of the angucycline glycosides (Fig. 5a, Table S5).

We have previously shown the applicability of quantitative proteomics combined with metabolomics to connect BGCs to the natural product(s) they specify^34^. Therefore, MS-based quantitative proteomic analysis was performed on total protein samples obtained from *Streptomyces* sp. MBT84 grown on MM agar plates with or without 100 µM catechol covered with a cellophane disc. After five days of growth, the biomass was harvested and snap-frozen in liquid nitrogen. Subsequent quantitative proteomics analysis was performed on three replicate samples per growth condition, yielding 1322 quantifiable proteins, of which 187 were significantly differentially expressed between catechol-grown and control cultures (FDR-adjusted *p*-value < 0.1; Fig. 5b). Proteins belonging to five of the 21 putative BGCs annotated by the antiSMASH algorithm were expressed under the chosen conditions. However, only the biosynthetic proteins of BGC4 were mostly significantly upregulated in catechol-grown cultures, which provided validation of the involvement of this BGC in the observed change in bioactivity induced by catechol (Fig. 5b, c). The minimal polyketide synthase (PKS) enzymes responsible for the generation of the initial angucycline or angucyclinone backbone^39^, namely two ketosynthase units (KSα and KSβ) and an acyl carrier protein, were not significantly differentially expressed (Fig. 5c).

To confirm that BGC4 was indeed responsible for the production of the angucycline glycosides, we knocked down transcription of the gene for the KSβ of the minimal PKS using CRISPR-dCas9 interference^40,41^. The knock-down was enforced from the CRISPRi construct pGWS1517 that expresses a spacer targeting the non-template strand of the gene for KSβ. Pigment production was strongly inhibited in the strain with reduced expression of KSβ, while no inhibition was seen in the control strain harboring a construct (pGWS1516) that targets the template strand (Fig. S4). Metabolomics analysis revealed that the production of the angucycline glycosides was significantly reduced when the expression of KSβ was inhibited (Fig. S4). All the evidence together proves that indeed BGC4 specifies the angucyclines.

### Expression of catechol-degrading enzymes counteracts antibiotic elicitation

Various microorganisms (e.g. *Pseudomonades*) enzymatically degrade aromatic compounds^42^. To establish whether catechol itself is essential for the eliciting effect, we expressed two enzymes in *Streptomyces* sp. MBT84 that degrade catechol. Catechol can be cleaved into *cis, cis*-muconic acid by catechol 1,2-dioxygenase (C12O) (*ortho*-pathway) or into 2-hydroxymuconic semialdehyde by catechol 2,3-dioxygenase (C23O) (*meta*-pathway) (Fig. S5)^42^. We wondered whether these degradation products would have a similar eliciting effect on angucycline production or whether the effect of catechol would disappear once degraded. To this end, we separately cloned the *catA1* gene encoding C12O from *Pseudomonas putida* KT2440 and the *xylE* gene encoding C23O from the promoter-probe vector pIJ4083^43^ onto the multi-copy vector pWHM3. The strong constitutive promoter SF14^44^ was used to drive expression. The resulting constructs pGWS1519 and pGWS1520 were introduced in *Streptomyces* sp. MBT84 via protoplast transformation, whereby the empty vector was used as a control. Of each recombinant strain (named MBT84-pWHM3, MBT84-C12O, MBT84-C23O) three independent transformants were tested for their ability to respond to catechol.

The transformants were grown for five days on MM agar plates with and without 100 µM catechol, and the metabolites were extracted as described above. While catechol induced a clear increase in yellow pigmentation in MBT84 harboring the control plasmid, such induction was no longer visible when MBT84-C12O was grown in the presence of catechol (Fig. S6A). In strain MBT84-C23O, the effect of catechol on the yellow pigmentation was strongly reduced, but in some transformants still some induction was observed. The visual assessment of antibiotic production was confirmed by detailed metabolomic analysis (Fig. S6B). Indeed, while catechol again elicited the production of metabolites in the control strain, catechol did not alter the metabolic profile of transformants expressing the catechol-degrading enzyme C12O or C23O. However, for the latter, the volcano plot showed a similar skewing to the upregulated side as the control, which suggests a reduced response.

To analyse the expression of the catechol dioxygenases and of BGC4 in the recombinant strains, we used quantitative proteomic analysis as described previously^45^. C12O and C23O were expressed individually in the respective recombinant strains (Fig. S6C). To study the protein levels of the gene products of BGC4 of the transformants or their parent with control plasmid, a heatmap of the log_2_ fold changes in expression between catechol and control was generated. In addition, violin plots were generated to show the distribution of the expression differences of BGC4 proteins (Fig. S6D). The small sample size and variability between the different transformants, did not allow for a thorough statistical analysis. However, while catechol induced a similar upregulation of the expression of BGC4 in MBT84-pWHM3 compared to WT, this pattern was no longer visible in MBT84-C12O and MBT84-C23O. These results indicate that catechol is the elicitor and not one of its degradation products.

## Discussion

Actinobacteria harbor many BGCs that are not or poorly expressed under laboratory growth conditions. A better understanding of the environmental signals that influence BGC expression as well as the regulatory pathways involved, will allow us to further explore actinobacterial specialized metabolism for therapeutic uses. Our work reveals catechol as elicitor of siderophore and antibiotic production in different streptomycetes. Specifically, catechol enhanced the expression of an angucycline BGC in *Streptomyces* sp. MBT84. Angucyclines constitute the largest group of aromatic polyketides and are well known for their structural diversity and their therapeutic potential as anticancer and antibiotic compounds^39^. Our recent discovery of the lugdunomycins and a range of novel angucyclines revealed that this extensively explored family of polyketides still has potential for drug discovery^13^. Indeed, the new compound galtamycin D (**8**) described in this study is another example that many angucyclines and derivatives are still to be discovered. Environmental cues may not only elicit the expression of (cryptic) biosynthetic pathways, but also of previously unseen branches of known pathways. Indeed, co-cultivation of the model streptomycete *Streptomyces coelicolor* A3(2) with *Aspergillus niger* elicited the production of GTRI-02, which is produced by the actinorhodin biosynthetic pathway^46^. The type II polyketide actinorhodin has been studied extensively by many laboratories for over 60 years, and the surprising discovery of an entirely new branch of its biosynthetic pathway underlines the importance of microbial interactions for drug discovery. Similarly, supplementation of catechol to growth conditions might offer a new strategy to further explore the immense chemical space of angucyclines and other polyketides. Preliminary experiments in our laboratory revealed many elicited mass features in the metabolome of other *Streptomyces* species grown in presence of catechol. Our data indicate that these mass features belong to non-ribosomal peptides and aminoglycoside antibiotics, which we are currently investigating. Thus, supplementation of catechol to growth media may allow for prioritization of bioactive metabolites for isolation from metabolite extracts which are often very complex mixtures.

To coordinate appropriate production of specialized metabolites, Actinobacteria have evolved a vast array of complex, multi-level regulatory pathways of which many remain to be elucidated. The identification of a signal that influences specialized metabolism can provide a first step towards the elucidation of a pathway, as was illustrated by the discovery that GlcNAc had a stimulating effect on antibiotic production of many *Streptomyces* sp.^23^. Elucidating the signal transduction pathway identified DasR as pleiotropic transcriptional repressor of several pathway-specific activators, including ActII‐ORF4 and RedD, whereby GlcN-6P acts as ligand for DasR^23^. We propose that the presence of catechol-containing molecules results in lower iron availability, which triggers siderophore production. However, the mechanism via which catechol impacts angucycline production in *Streptomyces*, remains yet unexplained. Our experiments show that the degradation of catechol will annihilate its effect. Heterologous expression of the catechol-cleaving enzymes C12O or C23O in *Streptomyces* sp. MBT84 strongly reduced its response to catechol. This strongly suggests that catechol is the elicitor and not one of its degradation products. Of the two enzymes, C23O had slightly less impact than C12O, whereby the latter fully counteracted the effect of catechol. Our proteomics experiments show that both enzymes are expressed well, and we anticipate that the difference may lie in the nature of their degradation products. C23O is responsible for the extradiol ring cleavage of catechol, producing 2-hydroxymuconic semialdehyde in which the 1,2 di-hydroxy architecture of catechol is preserved, while C12O does not maintain this scaffold. This could indicate that specific protein interactions are involved in the response to catechol and might also explain why *Streptomyces* sp. MBT84 only responds to catechol. Catecholamines are likely protonated at neutral pH, which could interfere with protein binding. The next step will be to elucidate the signal transduction pathway for catechol, and identify the key regulatory network it targets inside the cell.

Catechol impacts different types of specialized metabolites, as exemplified by its effect on the biosynthesis of angucyclines and siderophores. Production of the latter was enhanced by various catechol-containing plant- and animal-associated signaling molecules: the mammalian stress hormones epinephrine, dopamine, and norepinephrine (collectively known as catecholamines), their precursor levodopa, and the plant metabolite catechin. Interestingly, high throughput elicitor screening (HiTES) technology identified the plant-derived piceatannol as elicitor of the expression of a BGC for a cryptic NRPS^47^. Considering the data presented in our work, we hypothesize that this response might also be mediated through the catechol moiety that is present in piceatannol.

Siderophores are important mediators of interactions between members of microbial communities and the eukaryotic hosts they inhabit^18,48^. We show that plant and human-associated molecules with a catechol moiety increase siderophore production, resulting in decreased growth of *B. subtilis*. Potentially, catechol-mediated elicitation of siderophore production may influence the composition of microbial communities in the environment or within a eukaryotic host. The impact of our findings on the life of streptomycetes in the habitat is an intriguing question that requires further analysis.

Collectively, our results show that catechol, by itself and as molecular signature of signaling molecules, alters specialized metabolism in *Streptomyces*, likely via multiple modes of action. Understanding of the mechanisms involved is of great interest as they may offer tools to access silent and/or cryptic specialized metabolites from Actinobacteria.

## Methods

### Bacterial strains, growth conditions, and antimicrobial activity assay

All media and routine *Streptomyces* techniques have been described previously^49^. The bacterial strain collection used in this study was obtained from the Leiden MBT strain collection. *Streptomyces* sp. MBT42 and *Streptomyces* sp. MBT84 have been isolated from soil samples collected from Cheverny (France) and the QinLing Mountains (Shanxi province, China)^30^ respectively. *Bacillus subtilis* 168 and *Escherichia coli* ASD19^50^ were used as indicator strains for antimicrobial activity and were cultured in LB media at 37 °C.

Antimicrobial activity assays were conducted with 3 replicates using the double-layer agar method. Strains were spotted on minimal medium agar plates (MM) supplemented with 0.5% mannitol and 1% glycerol (w/v) as non-repressing carbon sources, and nutrient agar (NA) (Difco) plates, using a pin replicator. For individual testing of strains, 2 µL spore stock was manually spotted. Growth media were supplemented with 25 mM TES buffer and 100 µM of either (+)-catechin hydrate (Sigma-Aldrich, CAS# 225937-10-0), catechol (Sigma-Aldrich, CAS# 120-80-9), dopamine hydrochloride (Sigma-Aldrich, CAS# 62-31-7), (−)-epinephrine (+)-bitartrate salt (Sigma-Aldrich, CAS# 51-42-3), hydroquinone (Sigma-Aldrich, CAS# 123-31-9), levodopa (Sigma-Aldrich, CAS# 59-92-7), norepinephrine bitartrate monohydrate (MCE, CAS# 108341-18-0), phenol (VWR, CAS# 108-95-2), (R)-(−)-phenylephrine hydrochloride (Sigma-Aldrich, CAS# 61-76-7), tartaric acid (Sigma-Aldrich, CAS# 87-69-4), or tyramine hydrochloride (Sigma-Aldrich, CAS# 60-19-5).

After four days of incubation at 30 °C, plates were overlaid with soft LB agar (1.8% w/v agar) containing one of the indicator strains (2% v/v) pre-grown in liquid LB to exponential phase (OD_600_ = 0.4–0.6) and incubated overnight at 37 °C (±18 h). The following day, antibacterial activity was quantified by measuring the ratio between the surface area of the inhibition zone diameter and the spot diameter.

### Chrome azurol S (CAS) assay for siderophore detection

The medium for 1 L CAS was prepared according to the method of Schwyn and Neilands (1987)^51^ without the addition of nutrients: 60.5 mg CAS, 72.9 mg hexadecyltrimethyl ammonium bromide (HDTMA-Br), 30.24 g piperazine-1,4‐bis(2‐ethanesulfonic acid) (PIPES) and 10 mL of 1 mM FeCl_3_·6H_2_O in 10 mM HCl. Agarose (0.9% w/v) was used as gelling agent. The CAS agar solution was overlaid onto the spots of *Streptomyces* sp. MBT42. Following incubation overnight at 30 °C (±18 h) the plates were examined visually for halos.

### Bioactivity of crude extracts and metabolite profiling

*Streptomyces* sp. MBT84 was grown confluently on MM agar plates with and without 100 µM catechol (*n* = 6). After five days of growth, the agar plates were cut into small pieces, soaked overnight in ethyl acetate (EtOAc) to extract the metabolites, evaporated at room temperature and dissolved in MeOH to a final concentration of 20 mg/mL. 10 µL was spotted onto a sterile filter disc placed onto a soft agar layer inoculated with *B. subtilis* 168. As controls, 10 µL MeOH and 6 µL of 1 mg/mL ampicillin solutions were used. The following incubation overnight at 37 °C (±18 h), the plates were examined visually for halos.

For liquid chromatography-tandem mass spectrometry (LC-MS/MS) analysis, the dry extracts were dissolved in MeOH to a final concentration of 1 mg/mL. LC-MS/MS acquisition was performed using Shimadzu Nexera X2 ultra high-performance liquid chromatography (UPLC) system, with attached photodiode array detector (PDA), coupled to Shimadzu 9030 QTOF mass spectrometer, equipped with a standard electrospray ionisation (ESI) source unit, in which a calibrant delivery system (CDS) is installed. A total of 2 µL was injected into a Waters Acquity HSS C_18_ column (1.8 μm, 100 Å, 2.1 × 100 mm). The column was maintained at 30 °C, and run at a flow rate of 0.5 mL/min, using 0.1% formic acid in H_2_O, and 0.1% formic acid in acetonitrile (ACN) as solvents A and B, respectively. The gradient used was 5% B for 1 min, 5–85% B for 9 min, 85–100% B for 1 min, and 100% B for 4 min. The column was re-equilibrated to 5% B for 3 min before the next run was started. The PDA acquisition was performed in the range of 200–600 nm, at 4.2 Hz, with 1.2 nm slit width. The flow cell was maintained at 40 °C.

All the samples were analyzed in positive polarity, using data-dependent acquisition mode. In this regard, full scan MS spectra (*m/*z 100–1700, scan rate 10 Hz, ID enabled) were followed by two data-dependent MS/MS spectra (*m/z* 100–1700, scan rate 10 Hz, ID disabled) for the two most intense ions per scan. The ions were fragmented using collision-induced dissociation (CID) with fixed collision energy (CE 20 eV), and excluded for 1 s before being re-selected for fragmentation. The parameters used for the ESI source were: interface voltage 4 kV, interface temperature 300 °C, nebulizing gas flow 3 L/min, and drying gas flow 10 L/min.

### LC-MS based comparative metabolomics

Raw data obtained from LC-MS analysis were converted to mzXML centroid files using Shimadzu LabSolutions Postrun Analysis. The files were imported into Mzmine 2.53 for data processing^52^. Unless stated otherwise, *m/z* tolerance was set to 0.002 *m/z* or 10.0 ppm, RT tolerance was set to 0.05 min, noise level was set to 2.0E2 and the minimum absolute intensity was set to 5.0E2. Raw data were cropped to RT 0.5–12 min. Mass ion peaks were detected (positive polarity, mass detector: centroid) and their chromatograms were built using ADAP chromatogram builder^53^ (minimum group size in number of scans: 10; group intensity threshold: 2.0E2). The detected peaks were smoothed (filter width: 9), and the chromatograms were deconvoluted (algorithm: local minimum search; chromatographic threshold: 85%; search minimum in RT range: 0.05; minimum relative height: 1%; minimum ratio of peak top/edge: 2; peak duration: 0.03–2.00 min). The detected peaks were deisotoped (monotonic shape; maximum charge: 2; representative isotope: most intense). Peak lists from different extracts were aligned (weight for RT = weight for *m/z* = 20; compare isotopic pattern with a minimum score of 50%). Missing peaks detected in at least one of the samples were filled with the gap-filling algorithm (RT tolerance: 0.1 min). Among the peaks, we identified fragments (maximum fragment peak height: 50%), adducts ([M + Na]^+^, [M + K]^+^, [M + NH_4_]^+^, maximum relative adduct peak height: 3000%) and complexes (Ionization method: [M + H]^+^, maximum complex height: 50%). Duplicate peaks were filtered. Artifacts caused by detector ringing were removed (*m/z* tolerance: 1.0 *m/z* or 1000.0 ppm). The aligned peaks were exported to a MetaboAnalyst file.

In Excel, features that were not consistently present with an intensity higher than 8000 in all samples were removed from the file. In addition, all features that originate from the culture medium were removed by retaining only features with an average peak intensity of at least 50 times greater in the bacterial extracts than in the culture medium extracts. The resulting peak list was uploaded to MetaboAnalyst^35^ for statistical analysis. Log transformation with pareto scaling was applied to the data. Differences with a twofold change and an FDR-adjusted *p*-value < 0.1 were considered statistically significant (unless otherwise stated). Based on these criteria, volcano plots and heat maps were generated.

### MS/MS-based molecular networking and dereplication

MS/MS raw data (obtained from Shimadzu 9030 QTOF MS) were converted to a 32-bit mzML file using MSConvert (ProteoWizard) and a molecular network was assembled using the online Global Natural Product Social Molecular Networking (GNPS) tool^36^. Both the precursor ion and the MS/MS fragment ion mass tolerance were set to 0.02 Da. The minimum cosine score was set to 0.7 and the minimum matched peaks set to 5. The MSCluster algorithm was run with a minimum cluster size of 2 spectra. The spectra in the network were searched against the GNPS spectral libraries. For this, the precursor ion and the MS/MS fragment ion mass tolerance were set to 0.5 Da. Matches between network spectra and library spectra required a minimum score of 0.7 and at least 6 matched peaks. Cytoscape 3.5.1 was used for visualization of the generated molecular networks. The edge thickness was set to represent the cosine score, with thicker lines indicating higher similarity between nodes. LC-MS/MS data were deposited in the MassIVE Public GNPS data set (MSV000087784). The molecular networking job in GNPS can be found at https://gnps.ucsd.edu/ProteoSAFe/status.jsp?task=78cfa392f9c94ac0a110dc682a2d8e6f. The annotated MS/MS spectra were deposited in the GNPS spectral library for galtamycin C (CCMSLIB00006675753) and the new compound **8** which we designated as galtamycin D (CCMSLIB00006675754).

### Large-scale fermentation and isolation of metabolites 7 and 8

*Streptomyces* sp. MBT84 was grown on MM agar plates supplemented with 50 µM catechol, 1% glycerol and 0.5% mannitol at 30 °C for five days. Agar plates were cut into small pieces and soaked in EtOAc to extract metabolites as described earlier. The solvent was subsequently evaporated under reduced pressure at 40 °C to obtain 1.6 g crude extract. This extract was adsorbed onto 1.6 g silica gel (pore size 60 Å, 70–230 mesh, Sigma Aldrich), and loaded on a silica column, followed by gradient elution using mixtures of n-hexane, EtOAc, and MeOH. One of the fractions that eluted with 50% EtOAc: 50% n-hexane was combined with the fraction that eluted with 75% EtOAc: 25% n-hexane, and reconstituted in acetonitrile. This fraction was further purified using a Waters preparative HPLC system comprised of 1525 pump, 2707 autosampler, and 2998 PDA detector. The pooled fraction was injected into a SunFire C_18_ column (10 µm, 100 Å, 19 × 150 mm). The column was run at a flow rate of 12.0 mL/min, using solvent A (dH_2_O) and solvent B (acetonitrile), and a gradient of 70-100% B over 20 min to yield **7** (3.4 mg) and **8** (1.9 mg).

**Galtamycin D (8):** yellow amorphous powder; UV (LC-MS) λmax 222, 266, and 441 nm; HRESIMS *m/z* 579.1866 [M + H]^+^ (calcd for C_31_H_31_O_11_, 579.1861); ^1^H and ^13^C NMR data (Table S6).

### NMR measurements

NMR measurements for the purified compounds were recorded on Bruker Ascend 850 NMR spectrometer (Bruker BioSpin GmbH), equipped with a 5 mm cryoprobe. The samples were measured in DMSO-*d*_*6*_ in a 3 mm NMR tube through the use of an adapter. The spectra were referenced using the solvent residual peak and processed in MestReNova software.

### Genome sequencing, assembly, and annotation

*Streptomyces* sp. MBT42 and MBT84 were grown in 25 mL of YEME supplemented with 0.5% glycine and 5 mM MgCl_2_ and cultivated at 30 °C with 200 rpm shaking speed. Genomic DNA was isolated by phenol-chloroform extraction as described previously^49^. PacBio sequencing and assembly of *Streptomyces* sp. MBT42 was performed by Novogene (Novogene Europe, Cambridge, UK) using PacBio Sequel platform in continuous long reads mode. Raw sequences were demultiplexed with Lima v1.10.0, assembled using Flye v2.8.1^54^, and polished using Arrow v2.3.3. Genome sequencing of MBT84 was performed using Pacbio Sequel RSII at DNA link Sequencing Lab – South Korea. Raw sequences were demultiplexed with Lima 1.9.0 to produce CCS reads which were converted to fastq with bam2fastq 1.3.0. The assembly was performed with Flye 2.5^54^. The genomes of *Streptomyces* sp. MBT42 and MBT84 have been deposited at GenBank under accession numbers JAJNOJ000000000 and JAHTGP000000000. BGCs were identified using the genome mining tool antiSMASH 6.0^32^.

### Proteomics sample preparation

*Streptomyces* sp. MBT42 spores were spotted on NA plates supplemented with and without 100 µM catechin, dopamine, phenylephrine (*n* = 4). *Streptomyces sp*. MBT84 spores were spread on MM agar plates with and without 100 µM catechol using glass beads (*n* = 3). Plates were covered with cellophane before inoculation After five days incubation at 30 °C, biomass was scraped off and snap-frozen in liquid nitrogen, lysed in a precooled TissueLyser adaptor (Qiagen, The Netherlands) and proteins extracted using lysis buffer [4% SDS, 100 mM tris-HCl (pH 7.6), 50 mM EDTA]. The final peptide concentration was adjusted to 40 ng μL^−1^ using sample solution (3% acetonitrile, 0.5% formic acid) for analysis.

### Proteomics of catechol-grown cultures (proteomining)

For the analysis of the expression of biosynthetic enzymes in response to catechol, we applied natural product proteomining^34^. Samples were prepared from *Streptomyces* surface-grown cultures grown with or without catechol. Desalted peptide solutions were injected into Waters nanoAcquity UPLC system equipped with a Waters HSS T3 C_18_ (1.8 μm, 100 Å, 75 μm × 250 mm). A gradient from 1% to 40% acetonitrile in water (with added 0.1% FA) over 110 min was applied. Online MS/MS analysis was done using a Waters Synapt G2-Si HDMS mass spectrometer with a UDMS^E^ method set up as described previously^55^. [Glu1]-fibrinopeptide B was used as a lock mass compound and sampled every 30 s. Raw data from all samples were first analysed using the vender software ProteinLynx Global SERVER (PLGS, version 3.0.3, waters, USA). The resulting dataset was imported in ISOQuant version 1.8^55^ for label-free quantification and log_2_ fold changes were calculated. The mass spectrometry proteomics data have been deposited to the ProteomeXchange Consortium via the PRIDE^56^ partner repository with the dataset identifiers PXD029669, PXD030319, and PXD030484.

### Proteomics of *Streptomyces* sp. MBT84 expressing catechol dioxygenases

#### Heterologous expression of catechol dioxygenases

Constructs for the heterologous expression of catechol 1,2-dioxygenase (C12O) and catechol 2,3-dioxygenase (C23O) were constructed as follows The *catA1* gene encoding C12O was amplified from the genomic DNA of *Pseudomonas putida* KT2440 using the primer pair SF14_catA1_F and catA1_T0_R. The *xylE* gene encoding C23O was amplified from promoter-probe vector pIJ4083 using the primer pair SF14_xylE_F and xylE_T0_R. The forward primers contain the sequence of the strong constitutive promoter SF14^44^ including RBS and the reverse primers contain a t0 terminator sequence. The PCR products SF14-*catA1*-T0 and SF14-*xylE*-T0 were placed into the BamHI / XbaI site of the multi-copy vector pWHM3 to create vectors pGWS1519 and pGWS1520 respectively. These constructs were introduced into *Streptomyces* sp. MBT84 via protoplast transformation, whereby the empty vector was used as a control. An overview of the constructs and oligonucleotides is presented in Tables S1 and S2. For all experiments, the growth medium was supplemented with 10 µg/mL thiostrepton.

#### Analysis of the recombinant strains by quantitative proteomics

Quantitative proteomics was used to analyse the expression of catechol dioxygenases and the expression profile of BGC4 in recombinant strains of *Streptomyces* sp. MBT84 as described previously^45^. Briefly, the desalted peptide solution was separated on an UltiMate 3000 RSLCnano system (Thermo Scientific) set in a trap-elute configuration, coupled to QExactive HF (Thermo Scientific) mass spectrometer. The LC system used a Waters nanoEase M/Z Symmetry C_18_ trap column (5 µm, 100 Å, 180 µm × 20 mm) for peptide loading/retention, and Waters nanoEase M/Z HSS T3 C_18_ analytical column (1.8 µm, 100 Å, 75 µm × 250 mm) for peptide separation. The MS was operated in positive mode with data-dependent acquisition and default charge of 2. Raw LC-MS/MS files were analysed using MaxQuant software (v1.6.17.0)^57^ with label-free quantification (LFQ) method applied. Proteins were considered significantly altered in expression when FDR-adjusted *p* < 0.1 were obtained.

### CRISPRi technology

To knock down the expression of genes of the angucycline BGC, we applied CRISPRi RNA interference technology. The CRISPRi system was modified from pCRISPR-dCas9^41^ by expressing Cas9 from the constitutive *gapdh* promoter, using vector pSET152 that integrates at the øC31 attachment site on the *S. coelicolor* chromosome^58^. For this, a 20 nt spacer sequence was introduced into the sgRNA scaffold by PCR using forward primers KSβ_TF or KSβ_NT2F together with reverse primer SgTermi_R_B. The PCR products were cloned into pGWS1370^59^ via NcoI/BamHI restriction sites. The resulting constructs pGWS1516 (targeting template strand of KSβ, control) and pGWS1517 (targeting non-template strand of KSβ) were then introduced into *Streptomyces* sp. MBT84 via protoplast transformation^49^. An overview of the constructs and oligonucleotides is presented in Tables S1 and S2.

### Reporting summary

Further information on research design is available in the Nature Research Reporting Summary linked to this article.

## Supplementary information

Supplemental Material

Reporting Summary

## Data Availability

All datasets generated and/or analysed during the current study have been made publicly available. The metabolomics dataset are available in the GNPS repository, available at https://gnps.ucsd.edu/ProteoSAFe/status.jsp?task=78cfa392f9c94ac0a110dc682a2d8e6f. Sequence data have been deposited in Genbank with accession code JAHTGP000000000 and JAJNOJ000000000. In addition, the genomics and metabolomics data have been added to The Paired Omics Data Platform (project MSV000087784)^60^. Proteomics datasets have been deposited to the ProteomeXchange Consortium via the PRIDE partner repository with the dataset identifiers PXD029669, PXD030319, and PXD030484.
