# Supplementary material for: The ubiquitous catechol moiety elicits siderophore and angucycline production in Streptomyces
Source: Commun Chem. 2022 Feb 3;5:14. doi: 10.1038/s42004-022-00632-4 (PMC9814775; doi:10.1038/s42004-022-00632-4)
Supplement: Supplementary file 1 — Supplemental Material [file 42004_2022_632_MOESM1_ESM.pdf]

SUPPLEMENTARY INFORMATION

belonging to the manuscript

The ubiquitous catechol moiety elicits siderophore and angucycline production in *Streptomyces*

Doris A. van Bergeijk, Somayah S. Elsayed, Chao Du, Isabel Nuñez Santiago, Anna M. Roseboom, L. Zhang Victor J. Carrión, Herman P. Spaink, and Gilles P. van Wezel*

Institute of Biology, Leiden University, Sylviusweg 72, 2333 BE, Leiden, The Netherlands.

* author for correspondence. g.wezel@biology.leidenuniv.nl. Tel: +31 71 5274310.

Table S1. Overview of the primers used in this study

Primer name	sequence
SgTermi_R_B	CTAGGGATCCCAAAAAACCCCTCAAGACCCGTTTAGAGGCCCCAAGGGGTTATGCTAGTTACGCCTACGTAAAAAAAGCACCGACTCGGTGCC
KSβ_TF	CATGCCATGGGTTCGAGATGGGTGTGATCAGTTTTAGAGCTAGAAATAGC
KSβ_NT2F	CATGCCATGGGATGTCGCGGGGCAGTGTCCGTTTTAGAGCTAGAAATAGC
SF14_catA1_F	CATGGGATCCTAATGAGTTACGTAGACCTACGCCTTGACCTTGATGAGGCGGCGTGAGCTACAATCAATACTCGATTAGAATTCAAGGGAGAGAACATGACCGTGAAAATTTCCCAC
catA1_T0_R	CATGTCTAGATGGACTCACAAAGAAAAAACGCCCGGTGTGCAAGACCGAGCGTTCTGAACAATCAGCCCTCCTGCAACGCCCG
SF14_xylE_F	CATGGGATCCTAATGAGTTACGTAGACCTACGCCTTGACCTTGATGAGGCGGCGTGAGCTACAATCAATACTCGATTAGAATTCAAGGGAGAGAACATGAACAAAGGTGTAATGCGA
xylE_T0_R	CATGTCTAGATGGACTCACAAAGAAAAAACGCCCGGTGTGCAAGACCGAGCGTTCTGAACAATCAGGTCAGCACGGTCATGAA

Table S2. Overview of the plasmids used in this study

Plasmid	Description	
pWHM3	Unstable *E.coli* / *Streptomyces* shuttle vector with high copy number	[1]
GWS1370	pGWS1369 (pSET152 lacking its NcoI site) containing sgRNA scaffold (no spacer) and Pgapdh-dCas9	[2]
GWS1516	GWS1370 containing a spacer targeting the template strand of KSβ	This work
GWS1517	GWS1370 containing a spacer targeting the non-template strand of KSβ	This work
GWS1519	pWHM3 containing *catA1* behind SF14 promoter and a t0 terminator	This work
GWS1520	pWHM3 containing *xylE* behind SF14 promoter and a t0 terminator	This work

Table S3. Genomic features of *Streptomyces* sp. MBT42 and MBT84

	Streptomyces sp. MBT42	***Streptomyces* sp. MBT84**
Number of contigs	2	3
Largest contig	8,925,615	9,591,886
Total length	8,967,886	10,344,466
N50	8,925,615	9,591,886
CDS	7,748	9,713
rRNAs	21	18
tRNAs	85	90

Table S4. Biosynthetic gene clusters identified by antiSMASH 6.0 [3].

**Protocluster**	**Type**	**Most similar known cluster**
Contig_1		
1	RiPP-like	Informatipeptin (57%)
2	NAPAA	-
3 *	terpene	Hopene (92%)
4 *	T2PKS, oligosaccharide	Saquayamycin A (87%)
5	T3PKS	-
6	siderophore	Grincamycin (8%)
7 *	terpene	Geosmin (100%)
8	RiPP-like	-
9 *	siderophore	-
10	terpene	Albaflavenone (100%)
11 *	T2PKS	Spore pigment (83%)
12	siderophore	Desferrioxamine B (66%)
13	RiPP-like	-
14	ectoine	Ectoine (100%)
15	NAPAA	Chalcomycin (9%)
16	T3PKS	Herboxidiene (8%)
17	RRE-containing	
18	NRPS, betalactone	Cyclomarin D (17%)
19	Melanin	Melanin (42%)
20	NRPS, NAPAA, RiPP-like	Stenothricin (18%)
Contig_2		
21	Bacteriocin	-
Contig_3		
-		

* Expressed BGCs: ≥ 1 core biosynthetic protein was identified

Table S5. Comparison of BGC4 to saquayamycin cluster

Protein locus tag	Size (aa)	Putative function	Homologue in saquayamycin BGC0001769	%identity*

1	_08725	534	Multidrug-efflux transporter	sqnZ	98.50
2	_08730	246	Hypothetical protein	sqnAA	61.02
3	_08735	372	NADH oxidase	Not found	-
4	_08740	526	Multidrug resistance protein	sqnB	99.24
5	_08745	199	FMN-dependent NADPH-azoreductase	sqnC	100.00
6	_08750	227	Tetracycline repressor protein class E	sqnD	96.38
7	_08755	283	Hypothetical protein	sqnE	97.88
8	_08760	492	Anhydrotetracycline monooxygenase	sqnF	98.98
9	_08765	602	Tetracenomycin F2 cyclase	sqnBB	99.07
10	_08770	426	polyketide putative beta-ketoacyl synthase 1	sqnH	99.28
11	_08775	408	polyketide putative beta-ketoacyl synthase 2	sqnI	98.77
12	_08780	89	polyketide synthase acyl carrier protein	sqnJ	98.88
13	_08785	261	Putative ketoacyl reductase	sqnK	100.00
14	_08790	311	Putative polyketide cyclase	sqnL	99.04
15	_08795	665	Anhydrotetracycline monooxygenase	sqnM	98.95
16	_08800	404	putative MFS-type transporter EfpA	sqnN	99.26
17	_08805	430	Aclacinomycin-T 2-deoxy-L-fucose transferase	sqnG1	99.53
18	_08810	404	L-noviosyl transferase	sqnG2	99.26
19	_08815	193	dTDP-4-dehydrorhamnose 3,5-epimerase	sqnS1	100.00
20	_08820	376	L-noviosyl transferase	sqnG3	99.47
21	_08825	355	Glucose-1-phosphate thymidylyltransferase	sqnS2	99.72
22	_08830	327	dTDP-glucose 4,6-dehydratase	sqnS3	99.08
23	_08835	353	dTDP-6-deoxy-L-talose 4-dehydrogenase (NAD(P)(+))	sqnS4	97.79
24	_08840	434	GDP-perosamine synthase	sqnS5	99.54
25	_08845	254	dTDP-glucose 4,6-dehydratase	sqnS6	98.82
26	_08850	323	Hypothetical protein	sqnO	98.45
27	_08855	466	Hypothetical protein	sqnS7	99.57
28	_08860	318	Glucose--fructose oxidoreductase	sqnS8	98.11
29	_08865	241	4'-phosphopantetheinyl transferase psf-1	sqnCC	97.88
30	_08870	530	putative propionyl-CoA carboxylase beta chain 5	sqnP	99.62
31	_08875	221	Hypothetical protein	sqnDD	96.82
32	_08880	528	Aclacinomycin-N/aclacinomycin-A oxidase	sqnQ	99.81
33	_08885	145	Hypothetical protein	sqnEE	99.31
34	_08890	76	Hypothetical protein	Not found	-
35	_08895	254	Transcriptional regulatory protein YycF	sqnR	100.00
36	_08900	639	2-oxoglutarate oxidoreductase subunit KorA	sqnT	99.51
37	_08905	364	2-oxoglutarate oxidoreductase subunit KorB	sqnU	99.16

* sequences were aligned using protein blast

Table S6. ^{1}H and ^{13}C NMR data of 8 in DMSO-d_6 at 298 K

Position	δ_C, type	δ_H, mult. (J in Hz)
1	195.8, C	
2	50.4, CH_2	3.01, d (15.7)
		2.82, dd (15.7, 2.6)
3	75.8, C	
4	33.4, CH_2	3.57, dd (18.1, 2.4)
		2.94, dd (18.1, 2.9)
4a	138.3, C	
5	136.7, C	
6	114.8, CH	8.16, s
6a	130.3, C	
7	187.3, C	
7a	115.6, C	
8	158.1, C	
9	137.6, C	
10	133.8, CH	7.95, d (8.0)
11	119.2, CH	7.84, d (8.0)
11a	131.7, C	
12	187.4, C	
12a	117.1, C	
12b	160.5, C	
13	24.9, CH_3	1.45, s
8-OH		12.92, s
12b-OH		12.89, s
**Sugar A, β-D-olivose**		
1A	70.5, CH	5.01, dd (11.2, 2.2)
2A	35.9, CH_2	2.26, m
		1.62, m
3A	75.7, CH	3.88, m
4A	73.6, CH	3.52, dd (9.1, 9.1)
5A	73.5, CH	3.62, m
6A	17.4, CH_3	1.27, d (6.1)
**Sugar B, α-L-cinerulose B**		
1B	90.5, CH	5.23, d (2.7)
2B	70.9, CH	4.35, m
3B	39.8, CH_2	2.90, dd (17.4, 2.8)
		2.48, dd (17.4, 3.7)
4B	208.7, C	
5B	77.0, CH	4.71, q (6.8)
6B	16.1, CH_3	1.24, d (6.8)

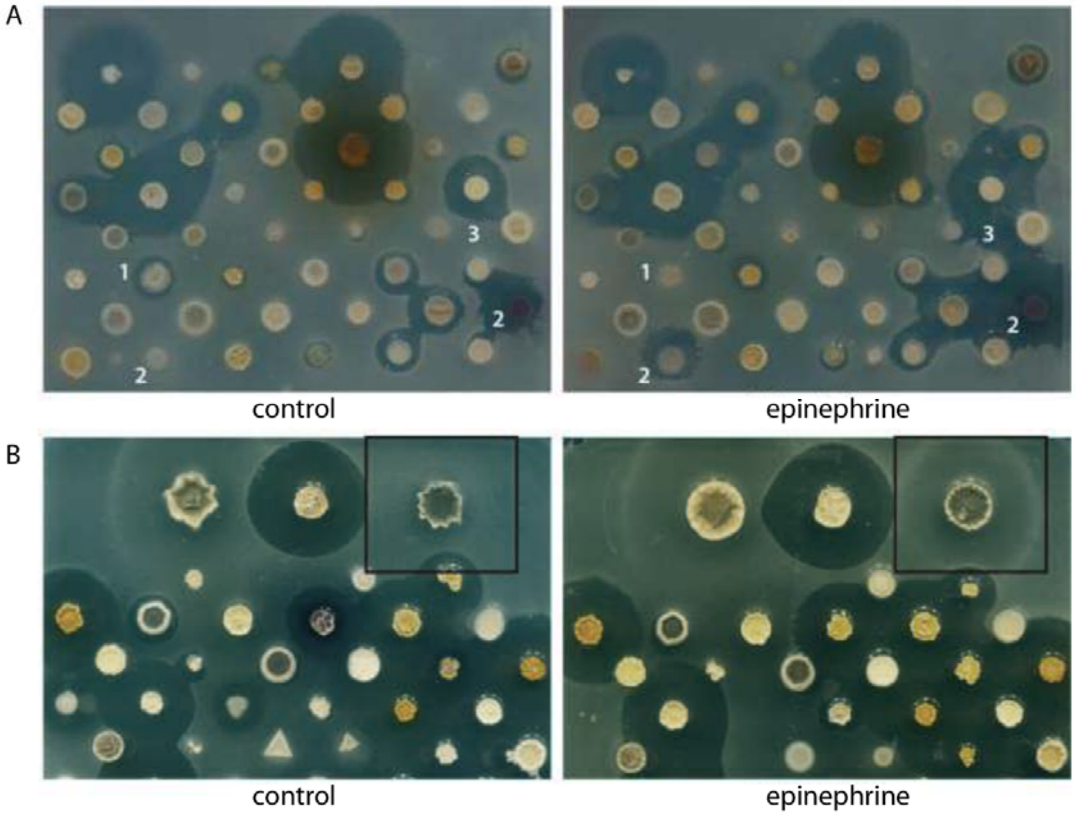

Figure S1. Epinephrine alters the bioactivity of different *Streptomyces*. A) On MM, both inhibition (1) and promotion (2) of antibiotic production in the presence of epinephrine is observed. Additionally, epinephrine can influence interactions between different strains illustrated by the change in halo shape (3) (n=3). B) On NA, Epinephrine elicits a semi-transparent halo surrounding *Streptomyces* sp. MBT42 (black square). (n=3).

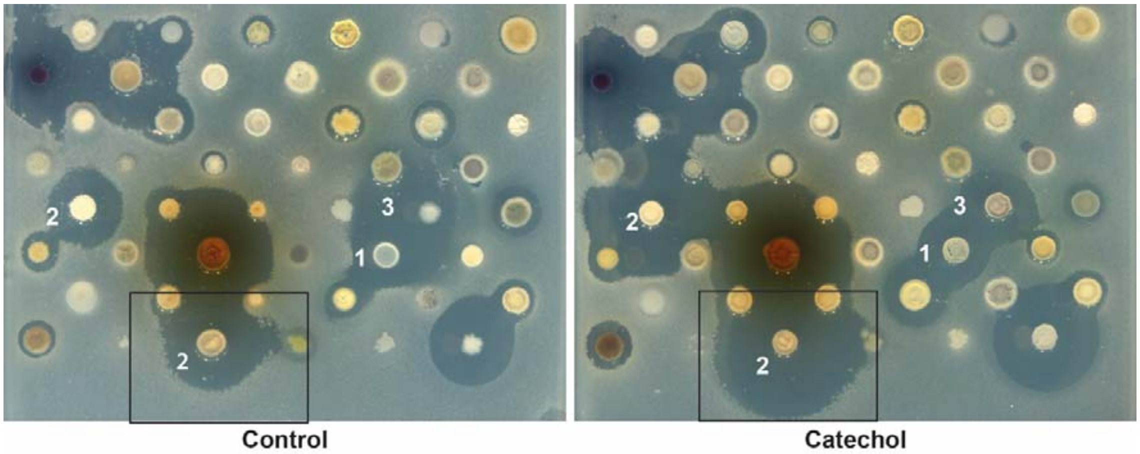

Figure S2. Catechol changes the bioactivity profile of different *Streptomyces*. *Streptomyces* sp. were spotted on MM with and without 100 µM catechol. Both inhibition (1) and promotion (2) of antibiotic production were observed. Additionally, we observed that catechol influenced interactions between different strains illustrated by the change in halo shape (3). Catechol significantly enhanced the bioactivity of *Streptomyces* sp. MBT84 (black square). (n=3)

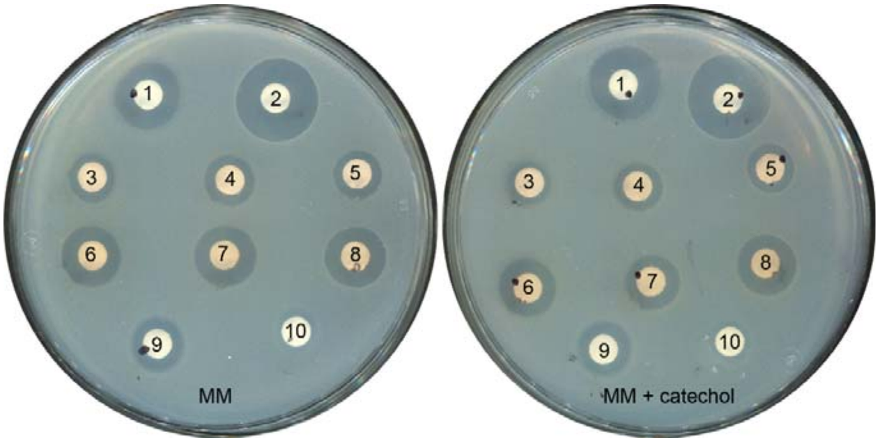

Figure S3. Addition of catechol to growth medium does not affect the susceptibility of *B. subtilis* 168 to different antibiotics and crude extracts of MBT84. MM glycerol mannitol agar plates with and without 100 µM catechol were overlaid with LB soft agar containing *B. subtilis* 168. Diffusion discs contain: 10 µL of (1) 0.5 mg/mL apramycin, (2) 0.5 mg/mL ampicillin, (3-5) crude extracts of MBT84 grown without catechol (20 mg/mL), (6-8) crude extracts of MBT84 grown in presence of catechol (20 mg/mL), (9) 1.5 mg/mL kanamycin, and (10) 5 mg/mL chloramphenicol. Experiment was conducted in duplicate with 2 replicates. No differences between the growth conditions were observed.

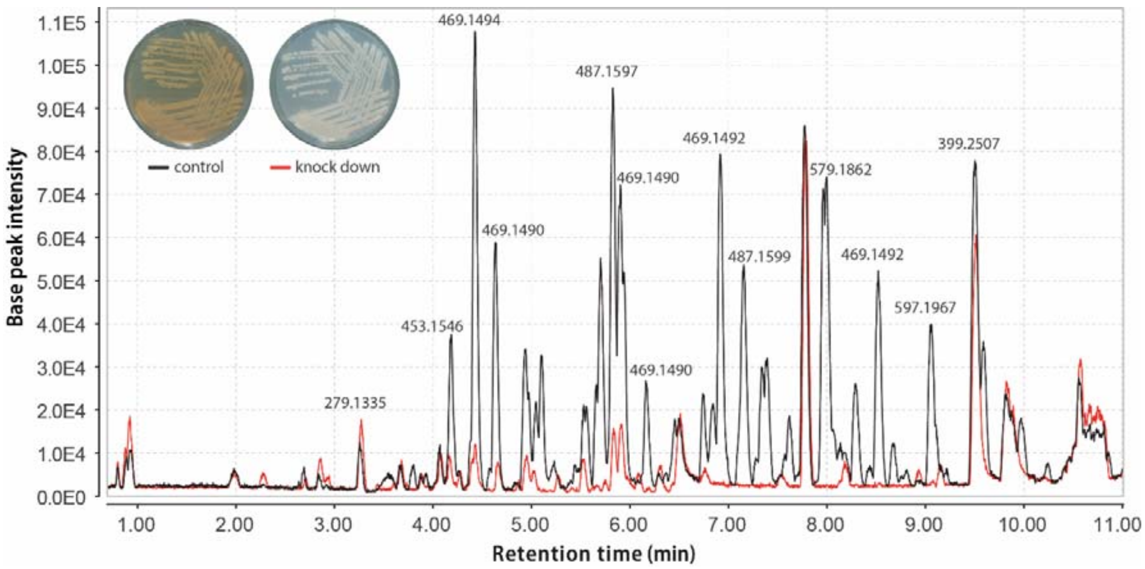

Figure S4. BGC4 specifies the angucyclines glycosides produced by *Streptomyces* sp. MBT84. Knock-down of the gene encoding KSβ of BGC4 in *Streptomyces* sp. MBT84 using CRISPRi resulted in almost complete inhibition of pigment production compared to the control strain harboring a construct that targets the template strand. The LC-MS chromatogram overlay of the crude extracts of the knock-down and control strains shows that inhibition of the expression of the KSβ resulted in decreased production of the majority of the metabolites. Three independent transformants were tested.

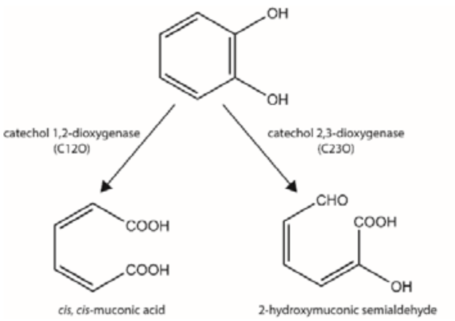

Figure S5. Degradation of catechol by catechol 1,2-dioxygenase and catechol 2,3-dioxygenase

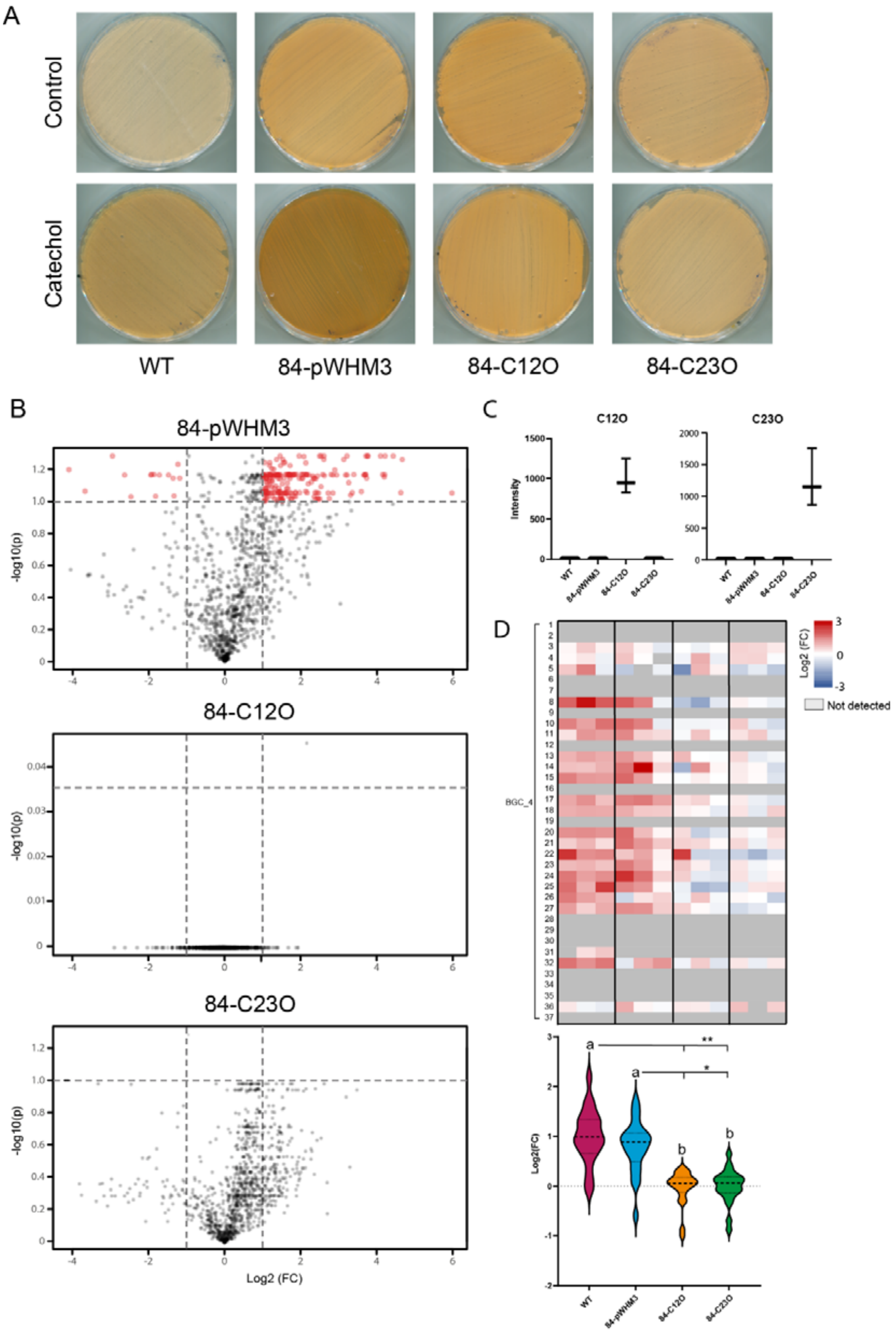

Figure S6. Heterologous expression of the catechol cleaving enzymes C12O and C23O resulted in a strong reduction of the eliciting effect of catechol. A) After five days of growth on MM with and without 100 μM catechol a clear increase in yellow pigmentation is

visible in the WT and MBT84-pWHM3 (empty plasmid control) strain. This increase is not visible when C12O and C23O are expressed. **B)** Volcano plot highlighting the changes in metabolite production of the different recombinant strains in response to catechol. While no difference in metabolite profile was found when C12O is expressed, the metabolite profile of MBT84-C23O follows a similar distribution as the control but none of the mass features are significantly upregulated in the catechol-grown cultures (fold change >2 and FDR-adjusted *p*-value < 0.1) (n=3). **C)** Protein abundance of the catechol dioxygenases in the WT and recombinant strains (n=3) **D)** Expression profile of BGC4 coding for the biosynthesis of angucycline glycosides in the WT and recombinant strains. The heatmap shows the $\log_2$ fold change of protein level of each transformant (or WT replicate) comparing samples from catechol-grown cultures and control cultures (n=3). The violin plots show the distribution of the $\log_2$ fold change of BGC4 expression in response to catechol (median = dashed line, quartiles = dotted line). Note that the empty vector control follows the same pattern as the WT strain, while this pattern is not visible when C12O and C23O are expressed. The average $\log_2$ fold changes in protein level of BGC4 in response to catechol were compared by one-way ANOVA, followed by a *post hoc* Tukey's HSD test. Similar letters indicate no significant difference (* = $p < 0.05$, ** $p < 0.01$).

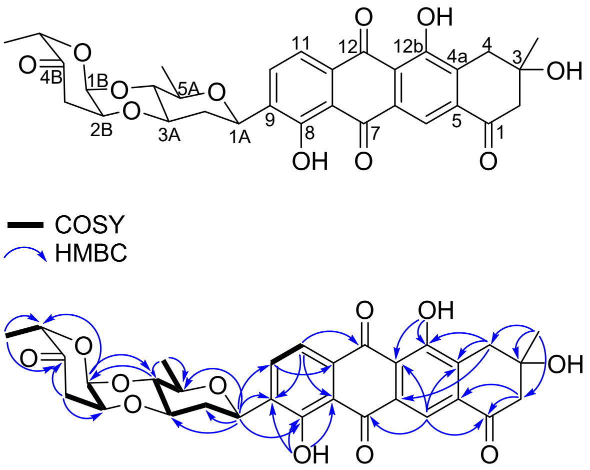

Figure S7. 2D structure (Top) and key COSY and HMBC correlations (bottom) of compound 8

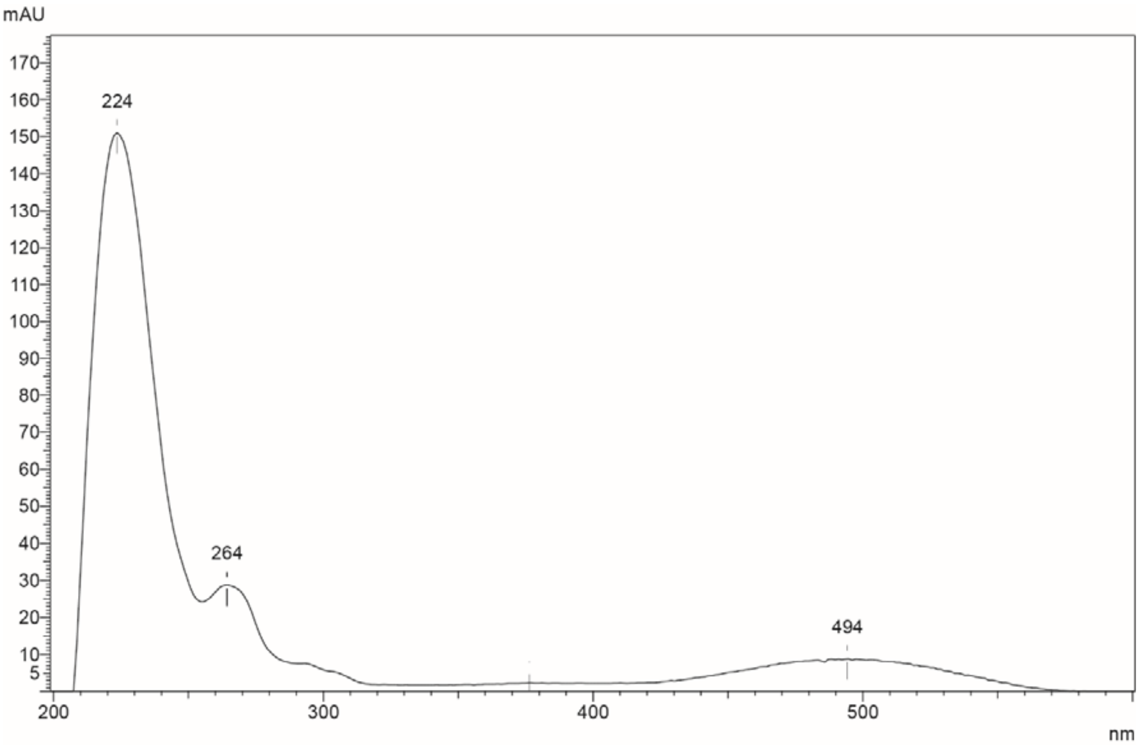

Figure S8. UV spectrum of 7

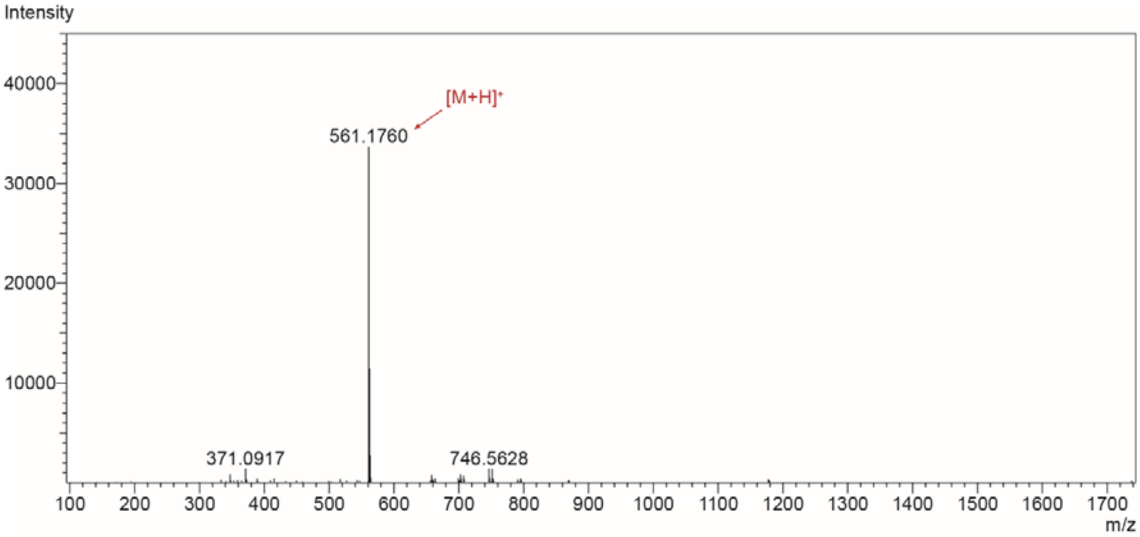

Figure S9. (+)-HR-ESI-MS spectrum of 7

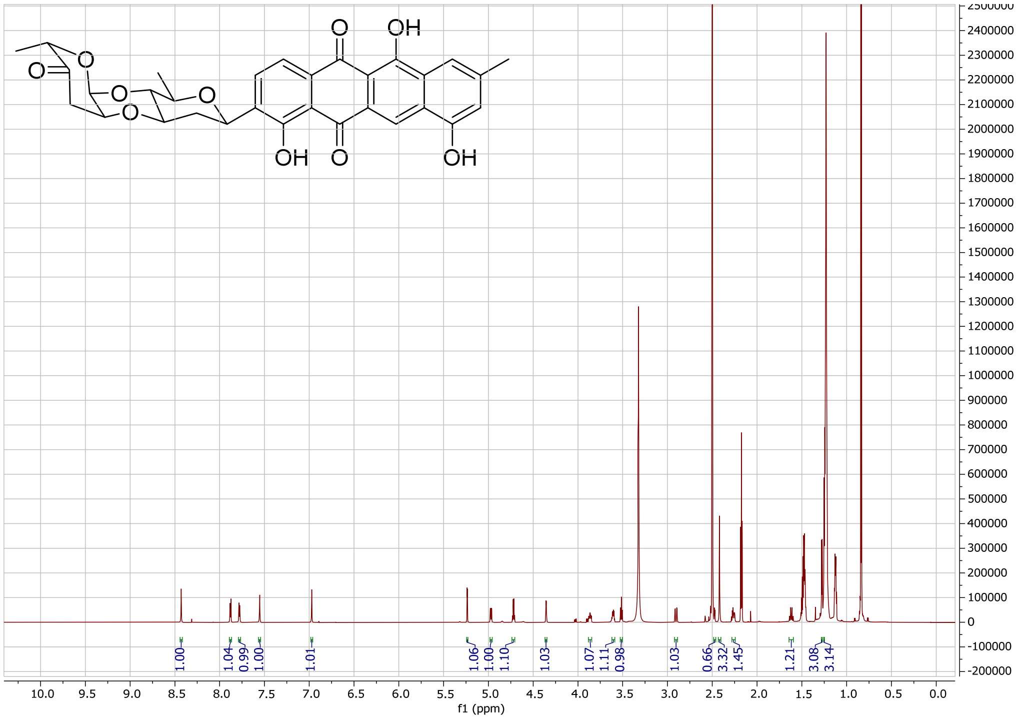

Figure S10. ^{1}H NMR spectrum of 7 (850 MHz, in DMSO-d_6)

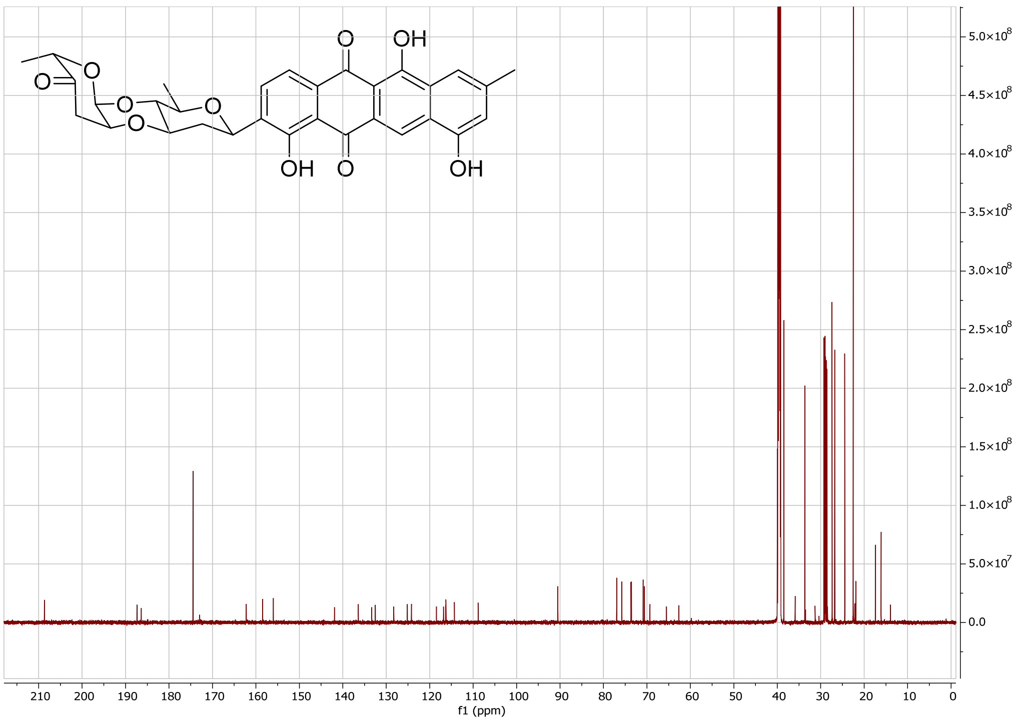

Figure S11. ^{13}C NMR spectrum of 7 (213 MHz, in DMSO-d_6)

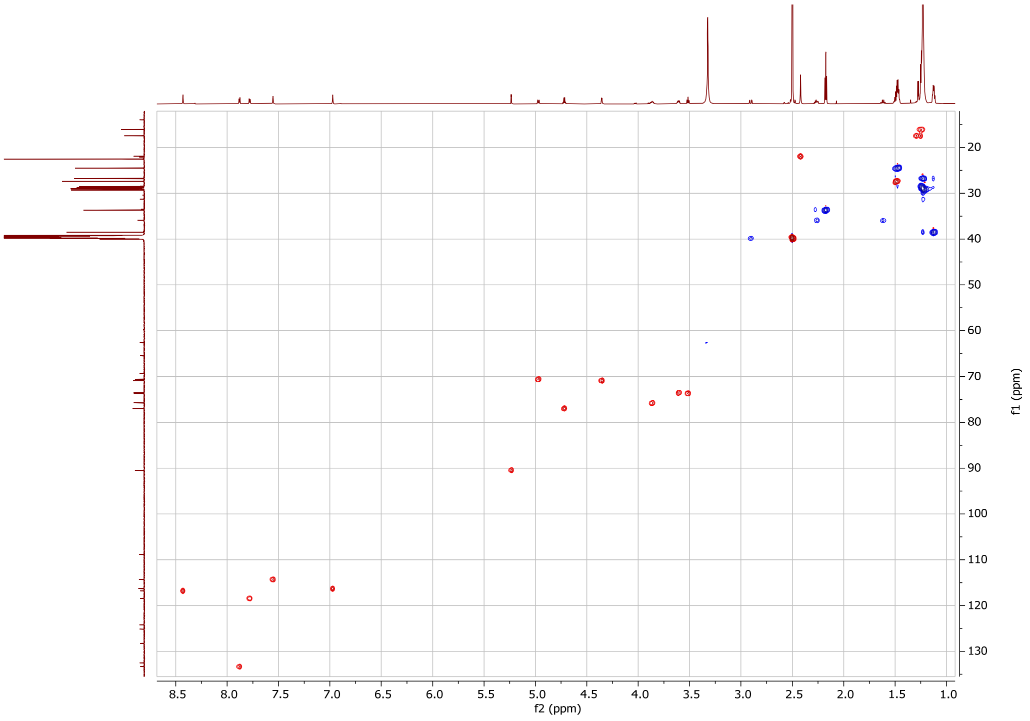

Figure S12. Multiplicity-edited HSQC spectrum of 7 (850 MHz, in DMSO-d_6)

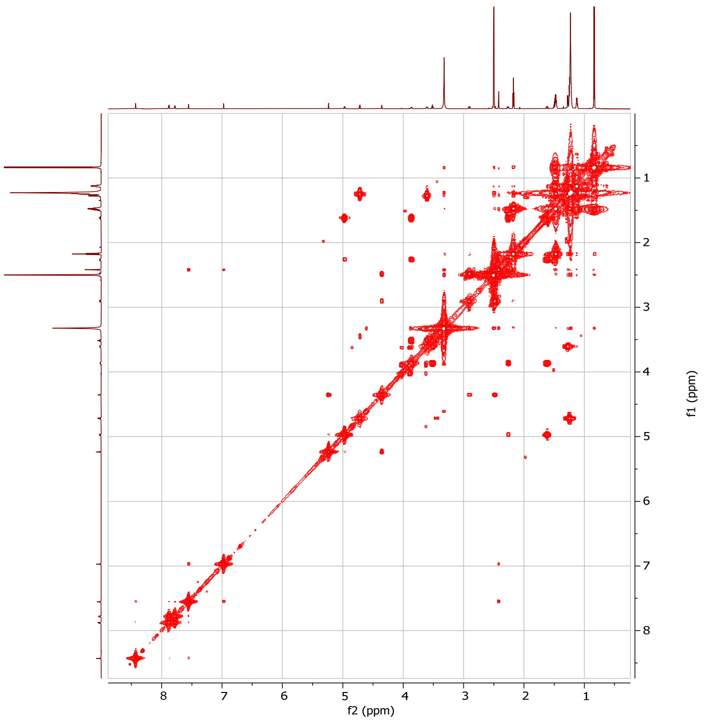

Figure S13. $^1H–^1H$ **COSY spectrum of 7 (850 MHz, in DMSO-d_6)**

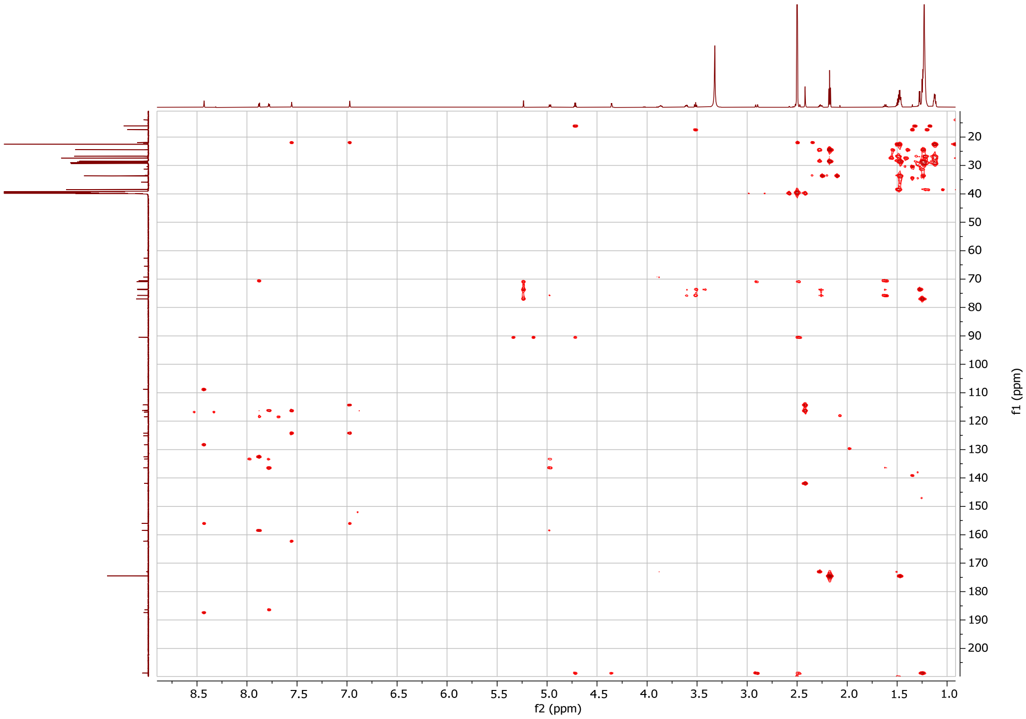

Figure S14. HMBC spectrum of 7 (850 MHz, in DMSO-d_6)

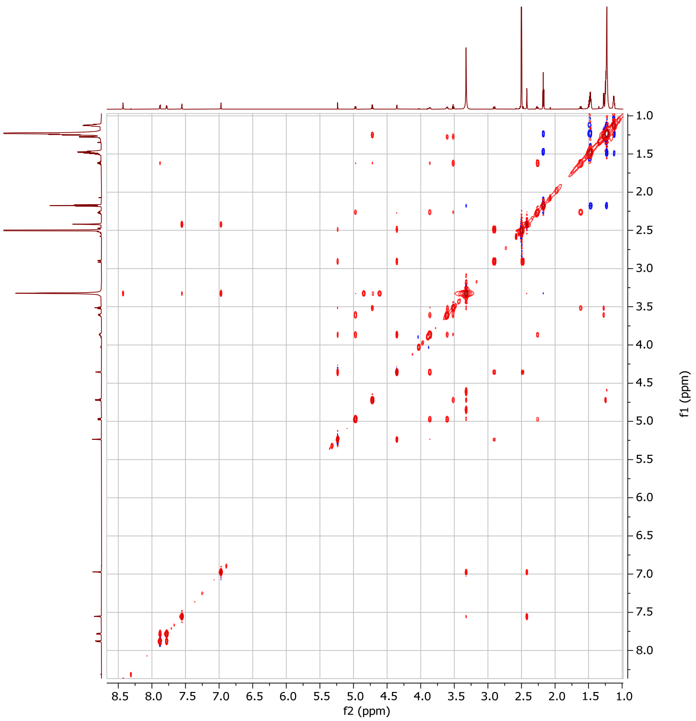

Figure S15. NOESY spectrum of 7 (850 MHz, in DMSO-d_6)

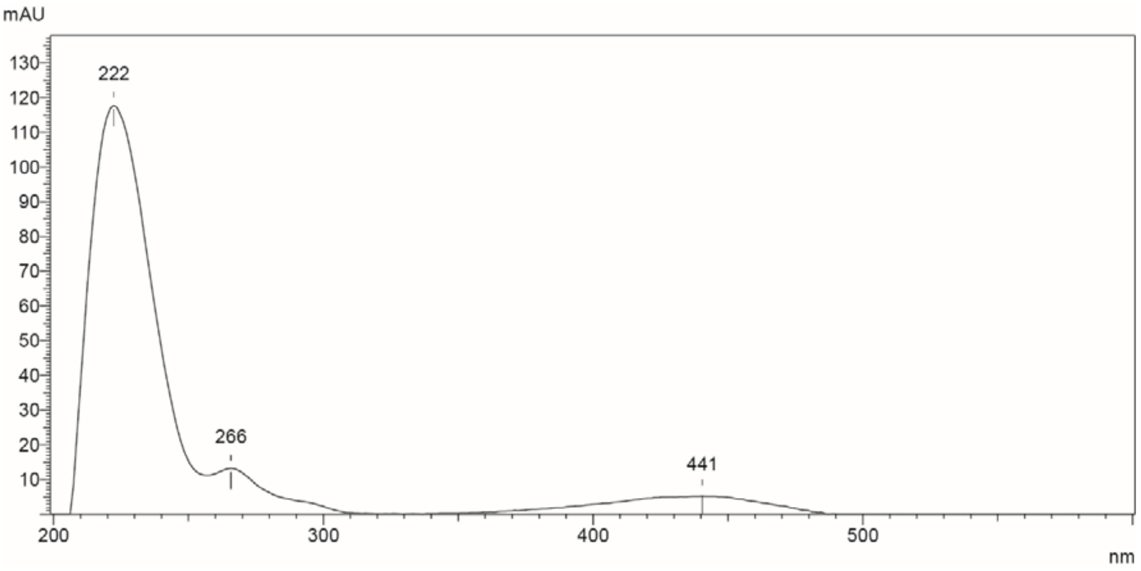

Figure S16. UV spectrum of 8

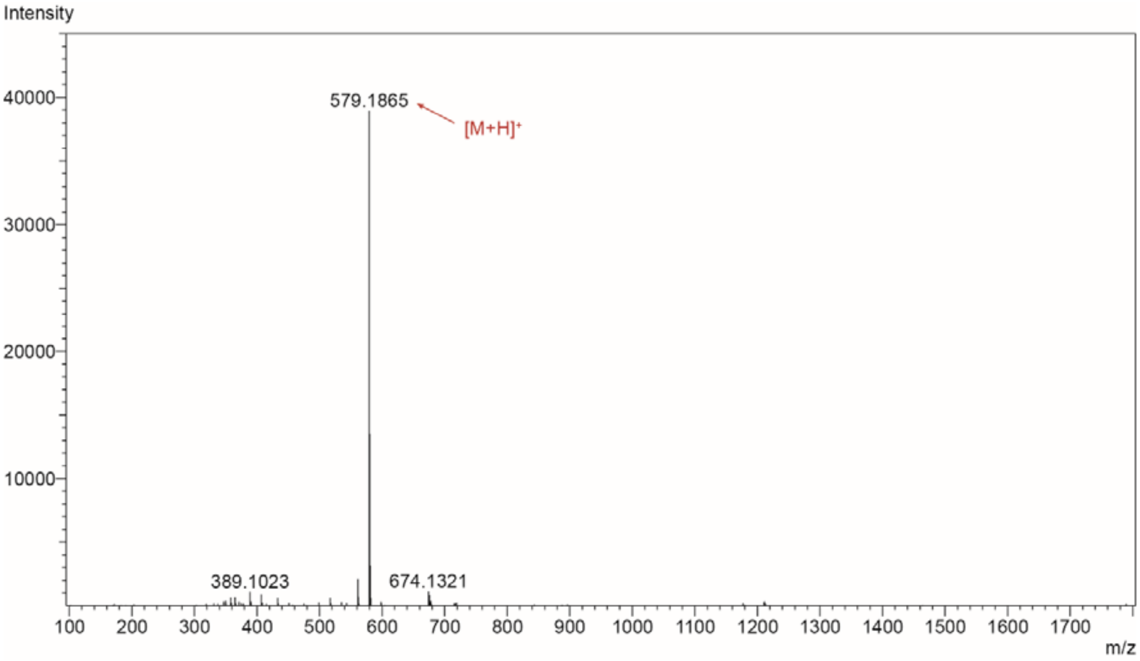

Figure S17. (+)-HR-ESI-MS spectrum of 8

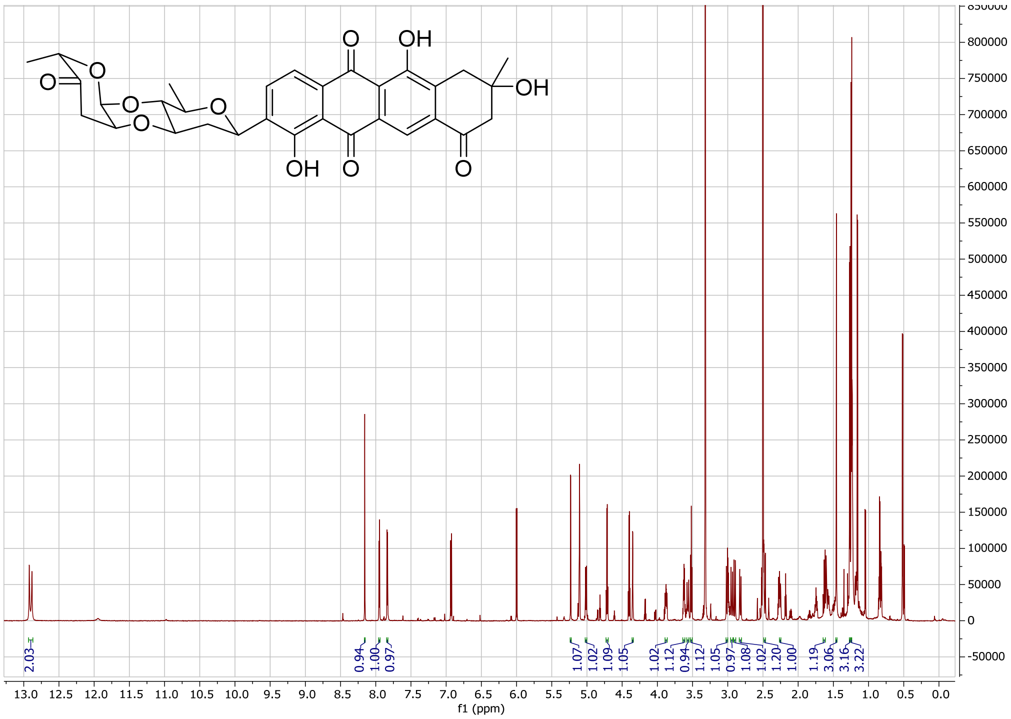

Figure S18. ^{1}H NMR spectrum of 8 (850 MHz, in DMSO-d_6)

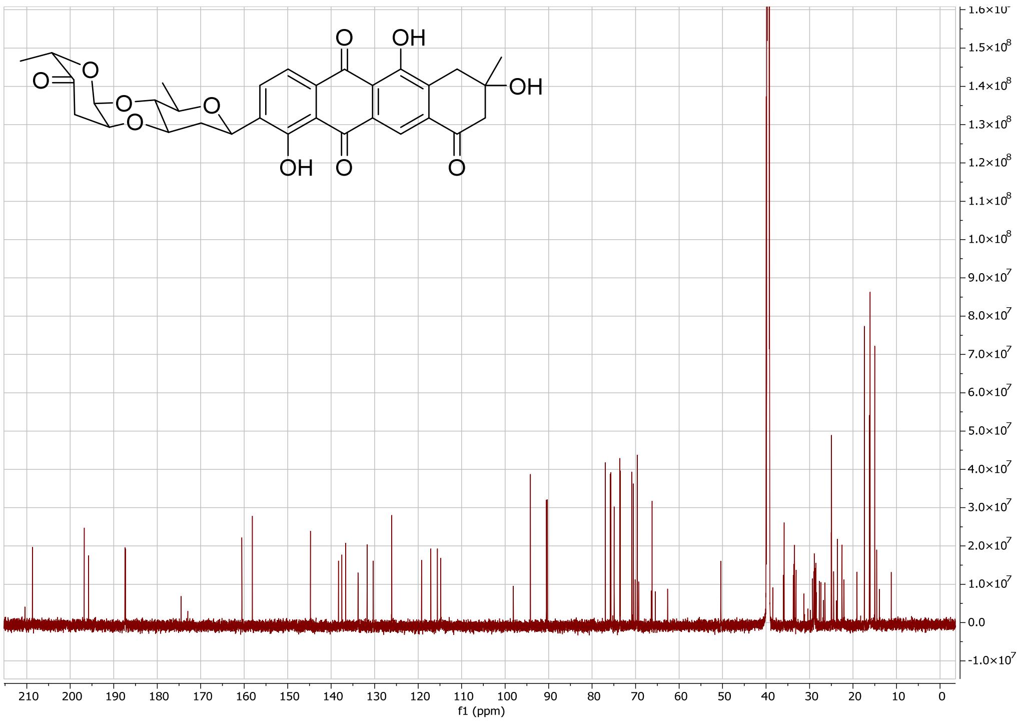

Figure S19. ^{13}C NMR spectrum of 8 (213 MHz, in DMSO-d_6)

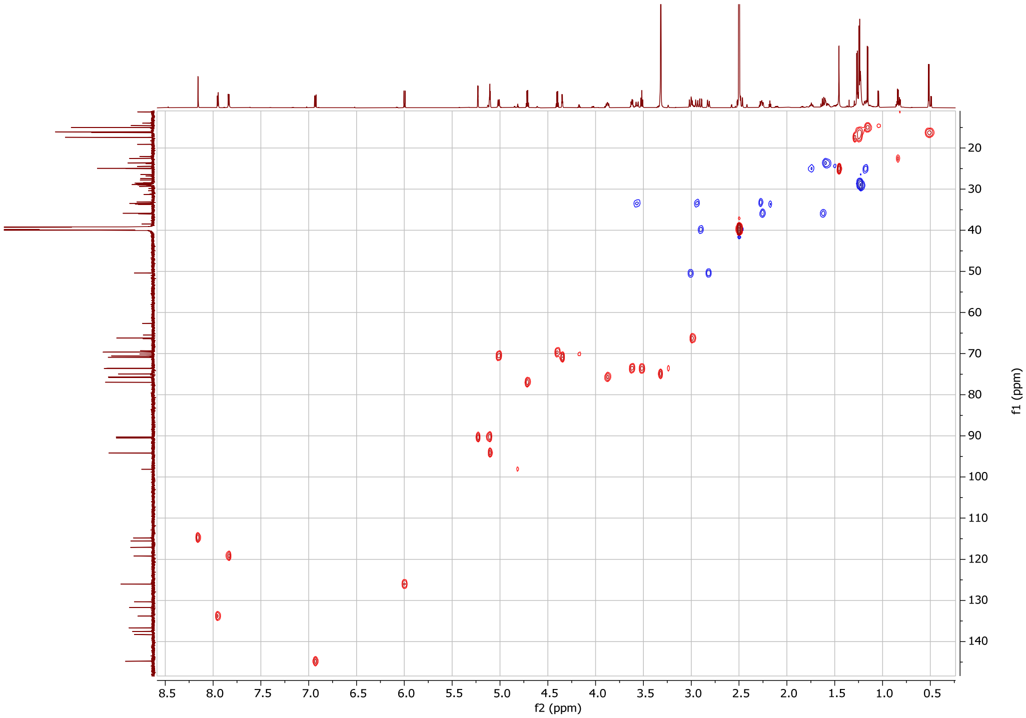

Figure S20. Multiplicity-edited HSQC spectrum of 8 (850 MHz, in DMSO-d_6)

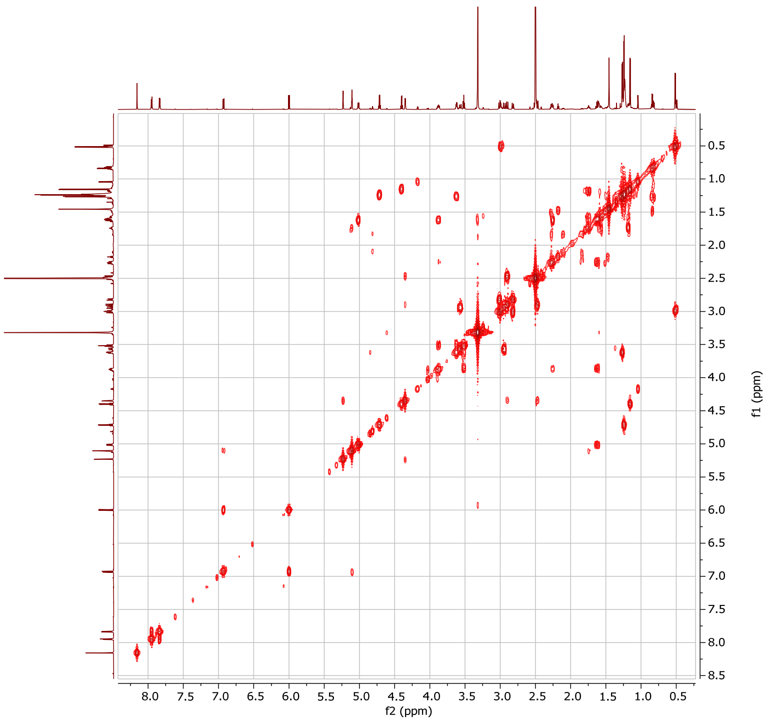

Figure S21. ^{1}H–^{1}H COSY spectrum of 8 (850 MHz, in DMSO-*d*$_6$)

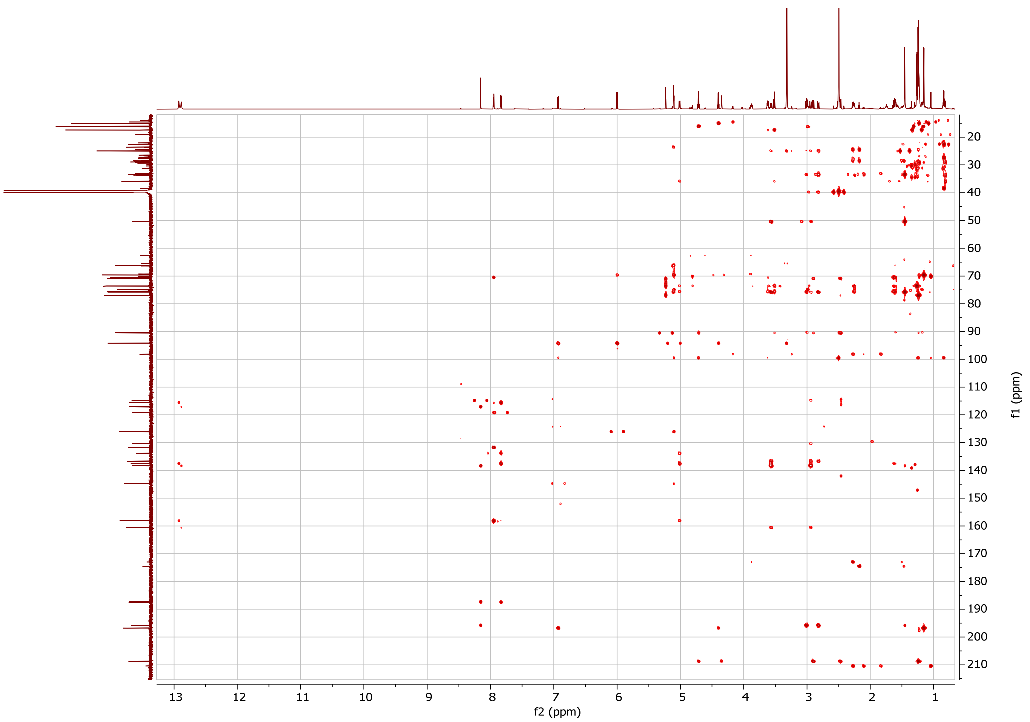

Figure S22. HMBC spectrum of 8 (850 MHz, in DMSO-*d*$_6$)

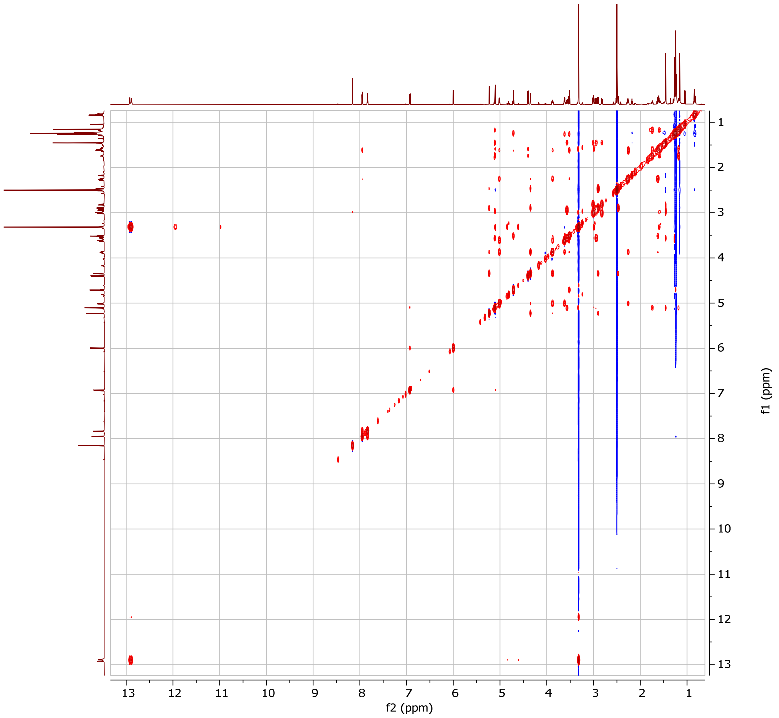

Figure S23. NOESY spectrum of 8 (850 MHz, in DMSO-d_6)

REFERENCES

1. Vara, J., Lewandowska-Skarbek, M., Wang, Y.G., Donadio, S. & Hutchinson, C.R. Cloning of genes governing the deoxysugar portion of the erythromycin biosynthesis pathway in *Saccharopolyspora erythraea* (*Streptomyces erythreus*). *J Bacteriol* **171**, 5872-81 (1989).
2. Zhang, L. et al. An Alternative and Conserved Cell Wall Enzyme That Can Substitute for the Lipid II Synthase MurG. *mBio* **12**, e03381-20 (2021).
3. Blin, K. et al. antiSMASH 6.0: improving cluster detection and comparison capabilities. *Nucleic Acids Res* **49**, W29-W35 (2021).
