# Supplementary material for: The ubiquitous catechol moiety elicits siderophore and angucycline production in Streptomyces
Source: Commun Chem. 2022 Feb 3;5:14. doi: 10.1038/s42004-022-00632-4 (PMC9814775; doi:10.1038/s42004-022-00632-4)
Supplement: Supplementary file 2 — Reporting Summary [file 42004_2022_632_MOESM2_ESM.pdf]

nature portfolio

Corresponding author(s): Gilles P. van Wezel

Last updated by author(s): Jan 7, 2022

Reporting Summary

Nature Portfolio wishes to improve the reproducibility of the work that we publish. This form provides structure for consistency and transparency in reporting. For further information on Nature Portfolio policies, see our Editorial Policies and the Editorial Policy Checklist.

Statistics

For all statistical analyses, confirm that the following items are present in the figure legend, table legend, main text, or Methods section.

n/a	Confirmed	
☐	☒	The exact sample size (*n*) for each experimental group/condition, given as a discrete number and unit of measurement
☐	☒	A statement on whether measurements were taken from distinct samples or whether the same sample was measured repeatedly
☐	☒	The statistical test(s) used AND whether they are one- or two-sided<br>*Only common tests should be described solely by name; describe more complex techniques in the Methods section.*
☒	☐	A description of all covariates tested
☐	☒	A description of any assumptions or corrections, such as tests of normality and adjustment for multiple comparisons
☐	☒	A full description of the statistical parameters including central tendency (e.g. means) or other basic estimates (e.g. regression coefficient) AND variation (e.g. standard deviation) or associated estimates of uncertainty (e.g. confidence intervals)
☐	☒	For null hypothesis testing, the test statistic (e.g. *F*, *t*, *r*) with confidence intervals, effect sizes, degrees of freedom and *P* value noted<br>*Give P values as exact values whenever suitable.*
☒	☐	For Bayesian analysis, information on the choice of priors and Markov chain Monte Carlo settings
☒	☐	For hierarchical and complex designs, identification of the appropriate level for tests and full reporting of outcomes
☒	☐	Estimates of effect sizes (e.g. Cohen's *d*, Pearson's *r*), indicating how they were calculated

Our web collection on statistics for biologists contains articles on many of the points above.

Software and code

Policy information about availability of computer code

Data collection	Genome sequencing data:Pacbio Sequel RSII, Lima 1.9.0, bam2fastq 1.3.0, Flye 2.5
Data analysis	the metabolomics data was analysed using Mzmine 2.53, MetaboAnalyst 5.0 and GNPS. NMR data was analysed using MestReNova. Proteomics data was analysed using ProteinLynx Global SERVER (PLGS, version 3.0.3, waters, USA), ISOQuant 1.8, MaxQuant V1.6.17.0, GraphPad Prism 9, RStudio v1.4.1717, and Excel. Genome sequences were annotated with Prokka and analysed with antiSMASH 6.0

For manuscripts utilizing custom algorithms or software that are central to the research but not yet described in published literature, software must be made available to editors and reviewers. We strongly encourage code deposition in a community repository (e.g. GitHub). See the Nature Portfolio guidelines for submitting code & software for further information.

Data

Policy information about availability of data

All manuscripts must include a data availability statement. This statement should provide the following information, where applicable:

- Accession codes, unique identifiers, or web links for publicly available datasets
- A description of any restrictions on data availability
- For clinical datasets or third party data, please ensure that the statement adheres to our policy

The metabolomics dataset generated and analysed during the current study are available in the GNPS repository, available at https://gnps.ucsd.edu/ProteoSAFe/status.jsp?task=78cfa392f9c94ac0a110dc682a2d8e6f.
Sequence data generated during this study have been deposited in Genbank with accession code JAHTGP000000000 and JAJNOJ000000000
Proteomics datasets generated and analysed during this study have been deposited to the ProteomeXchange Consortium via the PRIDE partner repository with the

dataset identifiers PXD029669, PXD030319, and PXD030484.

Field-specific reporting

Please select the one below that is the best fit for your research. If you are not sure, read the appropriate sections before making your selection.

☒ Life sciences ☐ Behavioural & social sciences ☐ Ecological, evolutionary & environmental sciences

For a reference copy of the document with all sections, see nature.com/documents/nr-reporting-summary-flat.pdf

Life sciences study design

All studies must disclose on these points even when the disclosure is negative.

Sample size	3 independent biological replicates were performed for all experiments.
Data exclusions	No data were excluded from analysis
Replication	A sample size of 3 independent biological replicates was chosen for all experiments. The results were consistent between repeated experiments.
Randomization	Transformants were selected randomly
Blinding	No blinding was performed in this study

Reporting for specific materials, systems and methods

We require information from authors about some types of materials, experimental systems and methods used in many studies. Here, indicate whether each material, system or method listed is relevant to your study. If you are not sure if a list item applies to your research, read the appropriate section before selecting a response.

Materials & experimental systems

n/a	Involved in the study	
☒	☐	Antibodies
☒	☐	Eukaryotic cell lines
☒	☐	Palaeontology and archaeology
☒	☐	Animals and other organisms
☒	☐	Human research participants
☒	☐	Clinical data
☒	☐	Dual use research of concern

Methods

n/a	Involved in the study	
☒	☐	ChIP-seq
☒	☐	Flow cytometry
☒	☐	MRI-based neuroimaging
